# Supplementary material for: (-)-Englerin A binding to human TRPC5 exposes an aromatic interaction network in channel activation
Source: Nat Commun. 2026 Apr 29;17:5259. doi: 10.1038/s41467-026-71840-y (PMC13263324; doi:10.1038/s41467-026-71840-y)
Supplement: Supplementary file 1 — Supplementary Information [file 41467_2026_71840_MOESM1_ESM.pdf]

Supplementary Information for:

(-)-Englerin A binding to human TRPC5 exposes an aromatic interaction network in channel activation

Sebastian A. Porav,[1,2,*] Alexandra Ptakova,[3,#] Claudia C. Bauer,[1,2,#] Kasia L. R. Hammond,[1,2] David J. Beech,[1] Viktorie Vlachova,[3] Stephen P. Muench,[2,4] and Robin S. Bon[1,2,*]

[1] *Leeds Institute of Cardiovascular and Metabolic Medicine, LIGHT Laboratories, University of Leeds, Leeds LS2 9JT, UK.*

[2] *Astbury Centre for Structural Molecular Biology, University of Leeds, Woodhouse Lane, Leeds LS2 9JT, UK.*

[3] *Department of Cellular Neurophysiology, Institute of Physiology, Czech Academy of Sciences, Videnska 1083, 142 00, Prague 4, Czech Republic.*

[4] *School of Biomedical Sciences, University of Leeds, Woodhouse Lane, Leeds LS2 9JT, UK.*

[#] *AP and CCB contributed equally*

** Correspondence should be addressed to RSB (r.bon@leeds.ac.uk) or SAP (s.porav@leeds.ac.uk).*

Table of Contents

Supplementary Notes 1-2……………..……………………………..……………………………p2

Supplementary Figures 1-15……………………………………………….…………………p4

Supplementary Tables 1-3……………………………………………………………………….p21

Supplementary Notes

Supplementary Note 1. Determination of TRPC5:EA^{PA} and TRPC5^{PA} structures.

Our initial TRPC5:EA^{PA} map (2.7 Å; C4 symmetry) displayed poorly resolved cytosolic domains, indicating structural flexibility of these domains. We tested different strategies to sort this heterogeneity in our data, including 3D classification, with and without symmetry expansion, focused classification, heterogenous refinement with different low-pass filtering and resolution limits, and various masking strategies (attempting to sort particles based on the presence or absence of EA). However, in all cases the different classes displayed similar EA density but poorly resolved ARDs/CCD. In contrast, 3D variability analysis (3DVA)[50] in CryoSparc[51] using 'clustering mode' resulted in two well-defined clusters depicting two TRPC5 states (**Figure 1a-f; Supplementary Figure 1a,b,d-g; Supplementary Figure 2**). In state 1 (TRPC5:EA^{PA}-S1), the ankyrin repeat domains (ARDs) are close to, and interact with, the coiled-coil domains (CCDs), resembling previous structures of TRPC5 in detergent (PDB 7E4T)[41] and lipid nanodics (PDB 8GVW)[43] (**Supplementary Figure 1d-g**). In state 2 (TRPC5:EA^{PA}-S2), the ARDs rotate counterclockwise (bottom view) while extending from the symmetry axis, similar to a state of human TRPC5 found in lipid nanodiscs (PDB 7X6C)[43] (**Supplementary Figure 1d-g**). The final 3D reconstructions, applying C4 symmetry, yielded maps of TRPC5:EA^{PA}-S1 and TRPC5:EA^{PA}-S2 at global resolutions of 2.5 Å (**Figure 1; Supplementary Figure 2**). The final map of 'apo' TRPC5^{PA} (depicting ARD state 2) was obtained at 2.4 Å resolution (C4 symmetry) (**Figure 1; Supplementary Figure 1; Supplementary Figure 2**). Further efforts to classify and sort particles from the 'apo' TRPC5^{PA} dataset did not reveal additional states.

Supplementary Note 2. Determination of six distinct TRPC5:EA structures from one data set.

To further test the effects of the addition of PA to our samples, including on local maps and on channel states, we decided to try an alternative sample preparation method. Instead of adding an EA/PA preparation to TRPC5 after purification, we maintained EA (100 µM) in all solutions throughout the sample preparation process, from cell lysis to grid making. This resulted in a 2.8 Å cryo-EM map (C4 symmetry; **Supplementary Figure 8**). We observed an unusual density that did not correspond to either EA or the resident lipid, suggesting partial occupancy (**Supplementary Figure 9**). Refining the map without applying symmetry (C1) resulted in a slightly lower resolution map (3 Å) displaying similar density in the EA binding sites, ruling out an artifact arising from symmetrisation.

We next performed 3DVA and visualised the output using 'simple mode' in CryoSPARC with 20 frames (**Supplementary Movie 1**). We noted a high degree of variability in the ARDs, consistent with the presence of the two states described for TRPC5:EA^{PA} (see above). Intriguingly, during examination of the area around the EA binding site, we observed a break in symmetry around the pore gate, shifting between C2 and C4 symmetry across different frames and structures (**Supplementary Movie 1**). Because the resolution was capped at 4 Å, this analysis did not provide further detail on EA/lipid stoichiometries in the individual structures. Therefore, we used 'clustering mode' to separate the data into the two previously described ARD states (see above). After refinement, we achieved maps with a resolution of ~3 Å (**Supplementary Figure 8; Supplementary Figure 9**). Although we refined these maps without imposing symmetry (C1), densities in the EA binding sites remained ambiguous.

Further processing with 3DVA allowed us to classify the data from each ARD state into three distinct classes (i.e. resulting in 6 unique maps), which were further categorised based on TRPC5:EA binding stoichiometry (**Supplementary Figure 8; Supplementary Figure 9; Supplementary Figure 12a-f**).

- State 1, full EA occupancy (TRPC5:$EA_{\mathbf{4:4}}$-S1; 2.8 Å; C4)
- State 1, two EA molecules per TRPC5 tetramer (TRPC5:$EA_{\mathbf{4:2}}$S1; 2.9 Å; C4 relaxed symmetry)
- State 1, mixed occupancy, 1-3 EA molecules per TRPC5 tetramer (TRPC5:$EA_{\mathbf{mix}}$-S1; 3.2 Å; C4 relaxed symmetry)
- State 2, two EA molecules per TRPC5 tetramer (TRPC5:$EA_{\mathbf{4:2}}$-S2; 3.0 Å; C4 relaxed symmetry)
- State 2, mixed occupancy of 1-3 EA molecules per TRPC5 tetramer (TRPC5:$EA_{\mathbf{mix1}}$S2; 3.1 Å; C4 relaxed symmetry)
- State 2, mixed occupancy of 1-3 EA molecules per TRPC5 tetramer (TRPC5:$EA_{\mathbf{mix2}}$-S2; 3.2 Å; C4 relaxed symmetry)

Additional efforts to separate the data for maps with mixed TRPC5:EA stoichiometry were unsuccessful.

Supplementary Figures and Tables

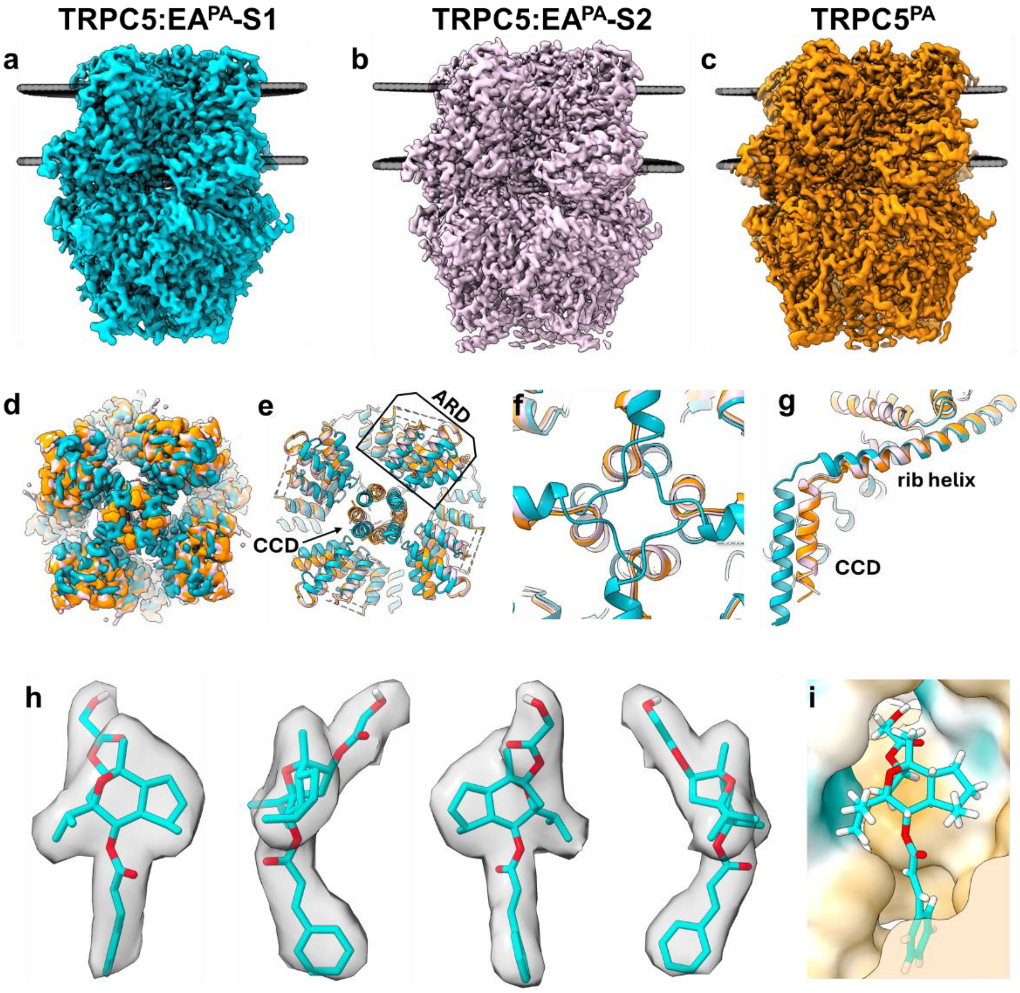

Supplementary Figure 1. Cryo-EM structures reveal multiple ARD states and the EA binding site of the human TRPC5 channel. a-c, cryo-EM maps of TRPC5:EA^{PA}-S1, TRPC5:EA^{PA}-S2 and TRPC5^{PA}. **d,e,** superimposition of cryo-EM maps and models of TRPC5:EA^{PA}-S1 (blue) and TRPC5:EA^{PA}-S2 (pink) and TRPC5^{PA} (orange) (bottom views) showing the main differences between ARD states. **f,** Close-up of the superimposed lower gates of TRPC5:EA^{PA}-S1 (blue) and TRPC5:EA^{PA}-S2 (orange) (bottom views). **g,** Side view of the superimposed CCD and rib helix of TRPC5:EA^{PA}-S1 (blue) and TRPC5:EA^{PA}-S2 (orange). **h,** Multiple viewing angles of EA fitted in the EM density of TRPC5:EA^{PA}-S1. **i,** Hydrophobic environment of the EA binding coloured by lipophilicity, with dark cyan being the most hydrophilic and goldenrod being the most lipophilic surface.

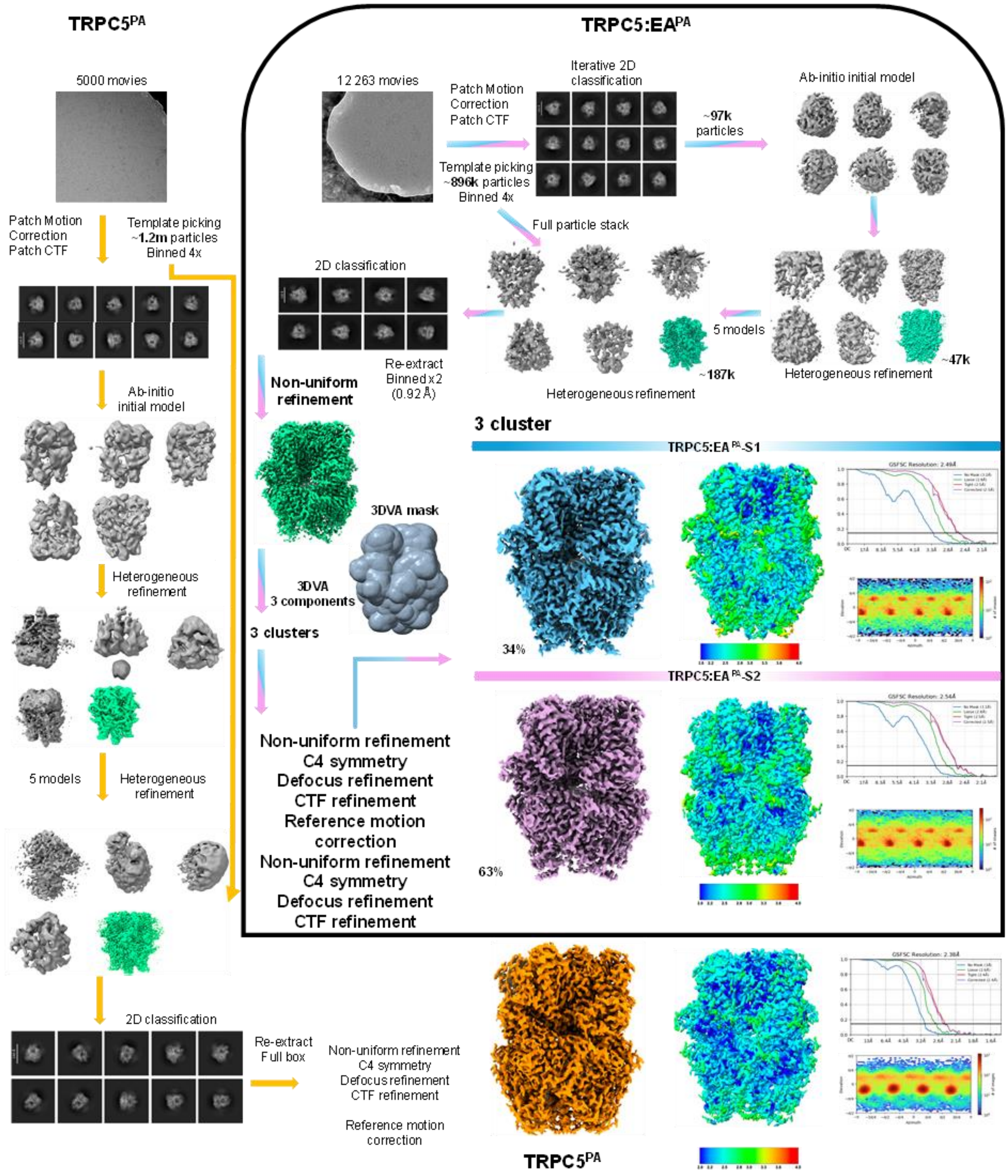

Supplementary Figure 2. Cryo-EM data processing workflow and map resolution of TRPC5:EA^{PA}-S1, TRPC5:EA^{PA}-S2 and TRPC5^{PA}.

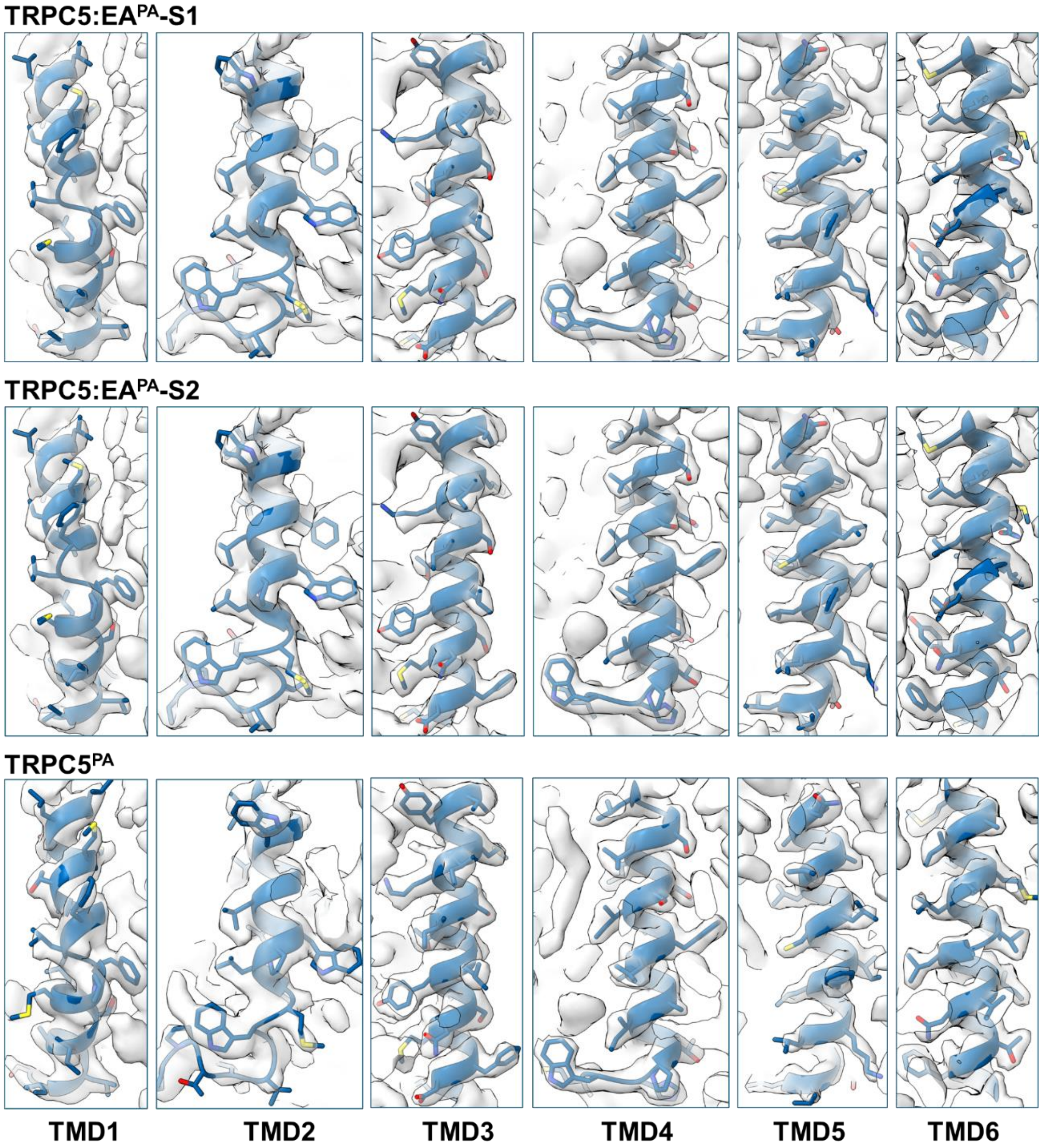

Supplementary Figure 3. **Data quality of TRPC5:EA^{PA}-S1, TRPC5:EA^{PA}-S2 and TRPC5^{PA} illustrated by the fit of the six transmembrane domains (blue) to the EM maps (grey).**

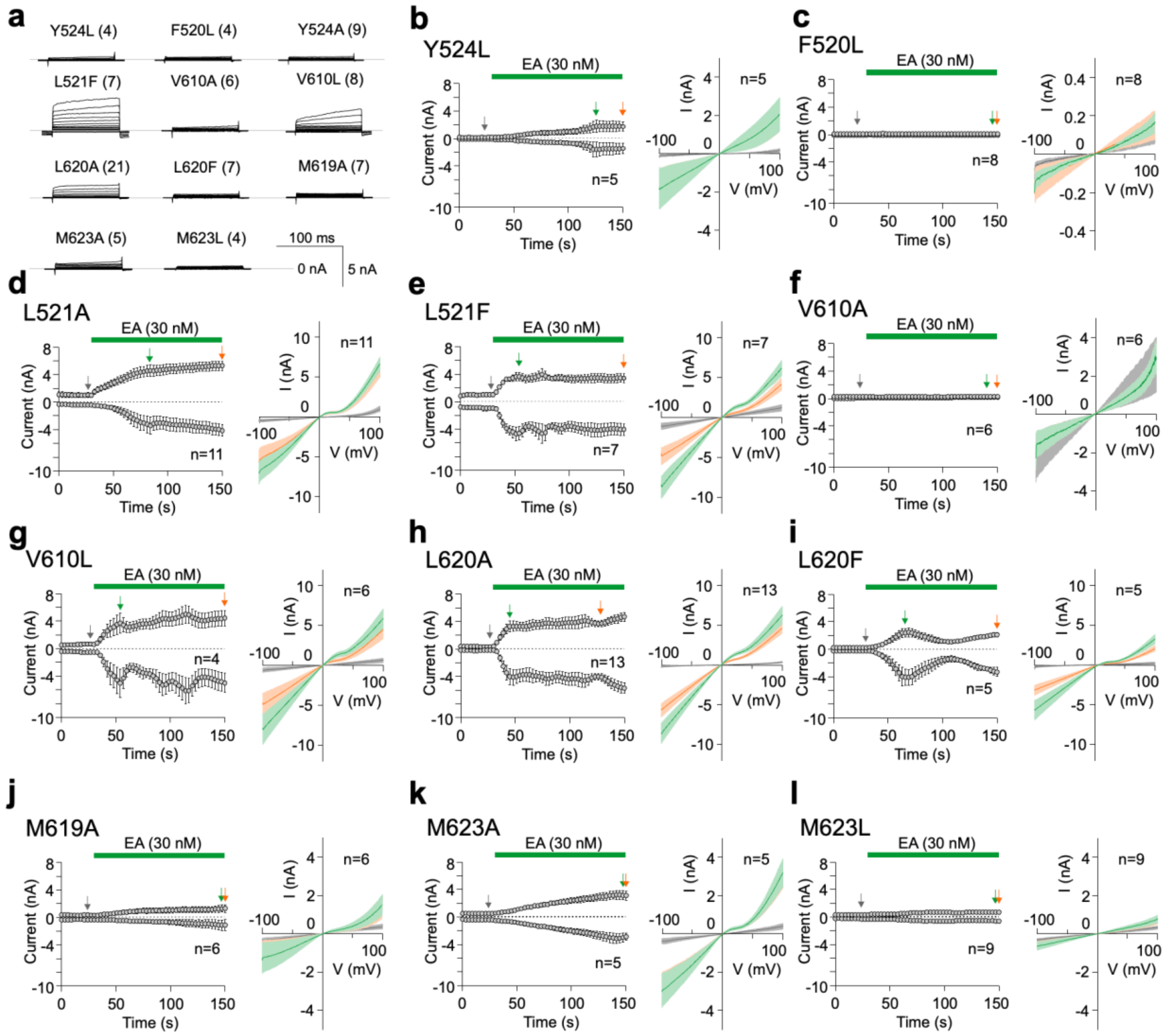

Supplementary Figure 4. Functional analysis of TRPC5 variants. a, Mean current trace in response to 100-ms voltage steps from -80 to +200 mV (20 mV increment) recorded from HEK293T cells expressing indicated TRPC5 constructs. The currents were recorded ~1 min after whole-cell formation in extracellular control solution. Numbers of cells (n) are indicated in parentheses. **b-l,** Time courses of average whole-cell currents elicited by 30 nM EA in HEK293T cells expressing indicated constructs of TRPC5. A ramp pulse from -100 mV to +100 mV was periodically applied from a holding potential of 0 mV every 3 seconds for 500 ms. Amplitudes were measured at -100 mV and +100 mV and the mean ± SEM was plotted as a function of time. Numbers of cells (n) are indicated. Right panels for each construct: mean current-voltage relations (coloured curves, ± SEM as lighter-coloured envelopes) are plotted for the currents measured at times indicated in the left panel by vertical arrows (grey at baseline, green at peak, and orange after 2-min exposure to EA). Source data are provided as a Source Data file.

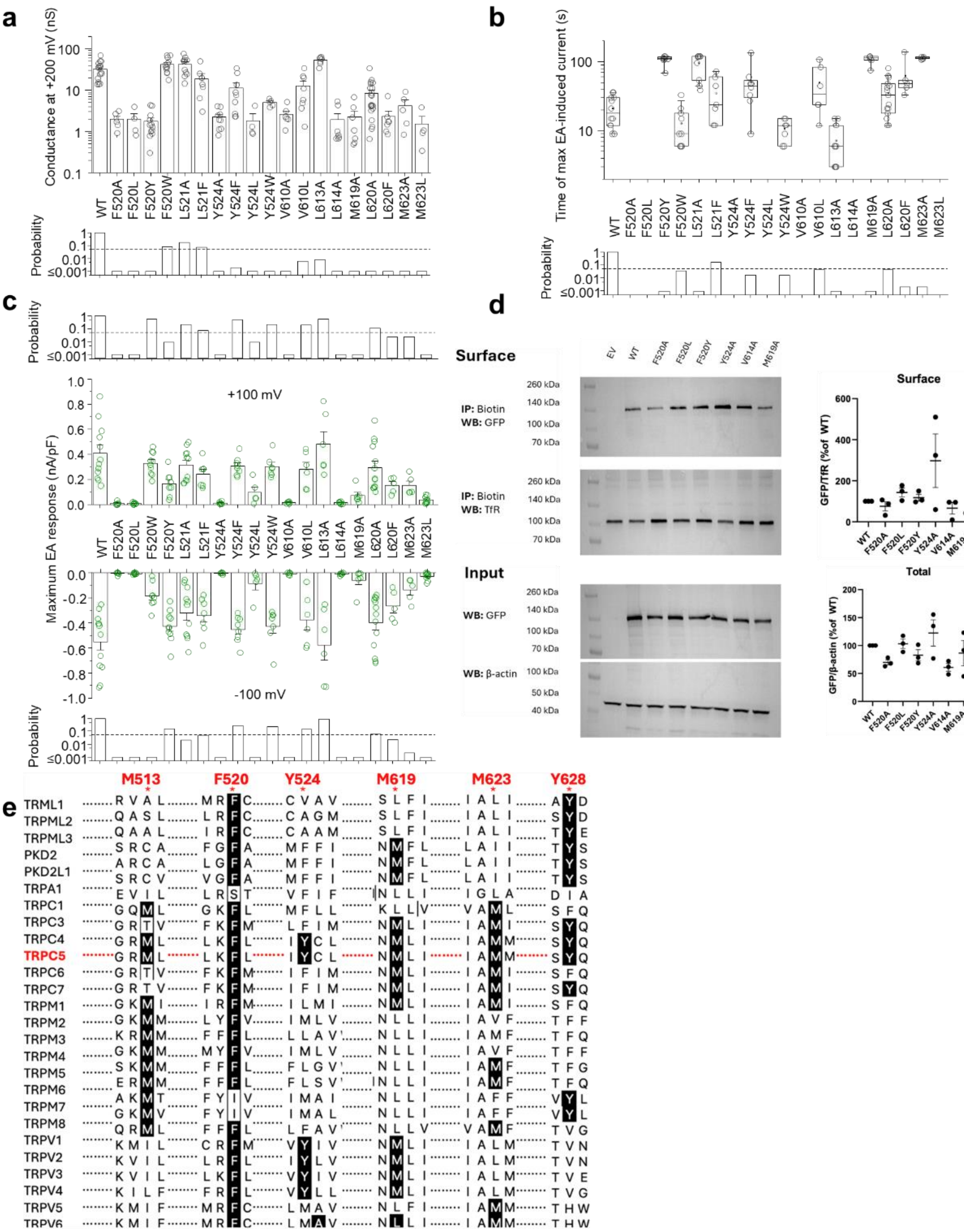

Supplementary Figure 5. Effects of mutagenesis of residues surrounding the EA binding site of TRPC5. a, Summary of effects of TRPC5 mutagenesis on voltage-induced TRPC5 activation, showing average currents at +200 mV (protocol shown in **Figure 4d**). Below, bar graph representing probabilities obtained from the Student's two-sided unpaired t-tests, performed to determine significance of differences between responses of wild-type and variants. The dashed horizontal line represents the statistical significance threshold (P < 0.05). **b,** Box plot of values of times to maximal responses mediated by the indicated constructs within 2 min of 30 nM EA application, measured at +100 mV. For each box, the centre line is the median value, square is the mean, the edges of the boxes are the 25th and 75th percentiles, and the lines extending from the boxes are the 5th and 95th percentiles (n ≥ 5). **c,** Bar graphs representing summarised current densities of maximal responses measured within 2 min of 30 nM EA application at +100 mV and -100 mV for indicated TRPC5 constructs. Above and below, bar graphs representing the probabilities obtained from the Student's two-sided unpaired t-tests, performed to determine significance of differences between responses of wild-type and variants. The dashed horizontal line represents the statistical significance threshold (P < 0.05). **d,** Surface biotinylation experiments of TRPC5-SYFP2 variants expressed in HEK293 cells. Biotinylated

surface proteins (upper blots) were pulled down using magnetic streptavidin beads and probed with antibodies against GFP (TRPC5-SYFP2) and Transferrin Receptor (TfR; loading control). Input samples were blotted with antibody against GFP (TRPC5-SYFP2) and β-actin (loading control) to confirm expression (lower blots). Blots were analysed using densitometry analysis and expression compared to control (WT TRPC5-SYFP2). Data are displayed as mean ± SEM. Data were analysed using one-way ANOVA with Dunnett's multiple comparison test to compare mutants to WT (control). For complete western blots, see **Supplementary Figure 15. e,** Conservation analysis of key TRPC5 residues involved in aromatic interactions that change upon EA binding. hTRPC5 residues were aligned with the sequences of other human TRP members in MEGA (v11.0.6) [Tamura et al. *Mol. Biol. Evol.* **38**, 3022–3027 (2021)]. Source data are provided as a Source Data file.

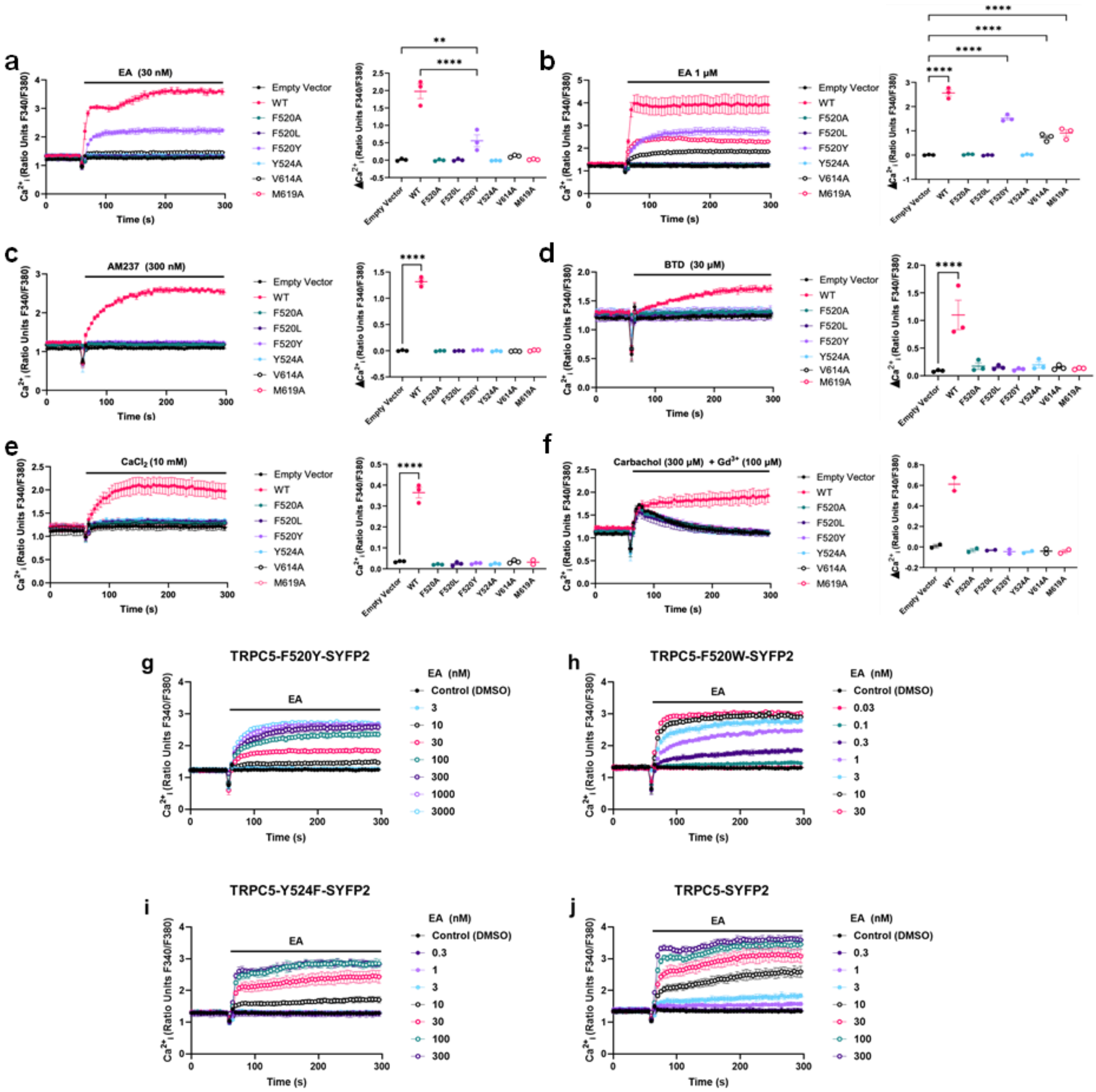

Supplementary Figure 6. Intracellular Ca^{2+} recordings of TRPC5-SYFP2 variants. a-f, Left, Representative traces from one 96-well plate (N = 6 technical repeats) each, showing increase in $[Ca^{2+}]_i$ of HEK293 cells expressing indicated TRPC5-SYFP2 variants, in response to different TRPC5 activators: 30 nM EA (a), 1 µM EA (b), 300 nM AM237 (c), 30 µM BTD (d), 10 mM extracellular $CaCl_2$ (e) or a combination of 300 µM carbachol and 100 µM $GdCl_3$ (f). Data are shown as mean ± SD. Right, mean responses (± SEM, n = 3 independent experiments) of experiments shown on the left of each panel, calculated by subtracting the basal levels (at 0-55 s) from the activator-induced responses (at 250-300 s). Data were analysed using one-way ANOVA with Dunnett's multiple comparison test to compare mutants to WT (control), and Šídák's multiple comparisons test (for a only) **g-j,** Representative traces from one 96-well plate (N = 6 technical repeats) each, showing concentration-dependent increases in $[Ca^{2+}]_i$ in response to indicated concentrations of EA in HEK293 cells expressing $TRPC5_{F520Y}$-SYFP2 (g), $TRPC5_{F520W}$-SYFP2 (h), $TRPC5_{Y524F}$-SYFP2 (i), and wild-type TRPC5-SYFP2 (j). Data are shown as mean ± SD. Corresponding concentration-response curves for (g-j) from 3 independent experiments (n = 3) are shown in **Figure 2l**. Source data are provided as a Source Data file.

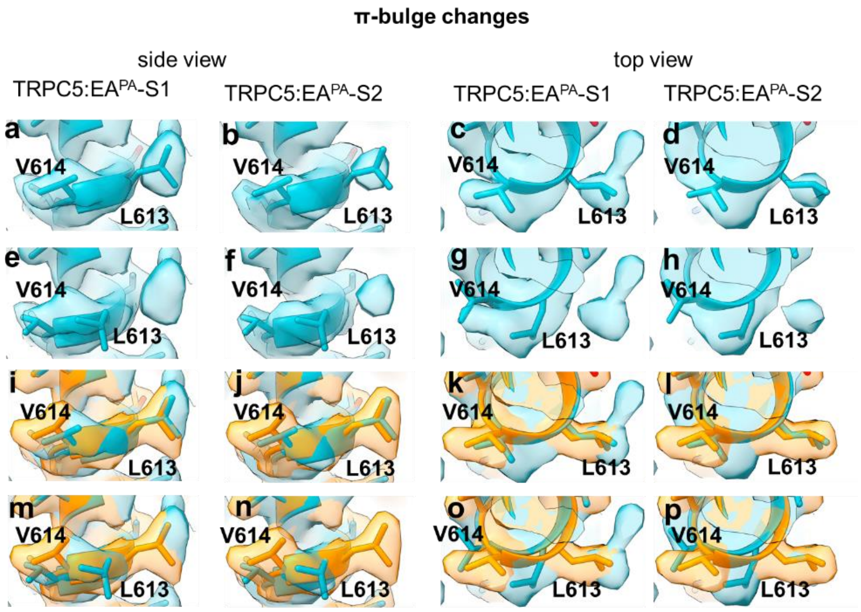

Supplementary Figure 7. EA binding induces flexibility of the TRPC5 π-bulge. a-h, Ambiguous density around the TRPC5 π-bulge allows fitting of different rotameric position for L613 and V614 into the EM maps of TRPC5:EA^{PA}-S1 (a,c,e,g) and TRPC5:EA^{PA}-S2 (b,d,f,h), shown from the side (a,b,e,f) and top (c,d,g,h). **i-p,** Overlays of the maps and models of the TRPC5 π-bulge in TRPC5:EA^{PA}-S1 and TRPC5:EA^{PA}-S2 and the map and model of TRPC5^{PA} (orange), illustrating the differences in map certainty and suggesting EA-induced flexibility of this region.

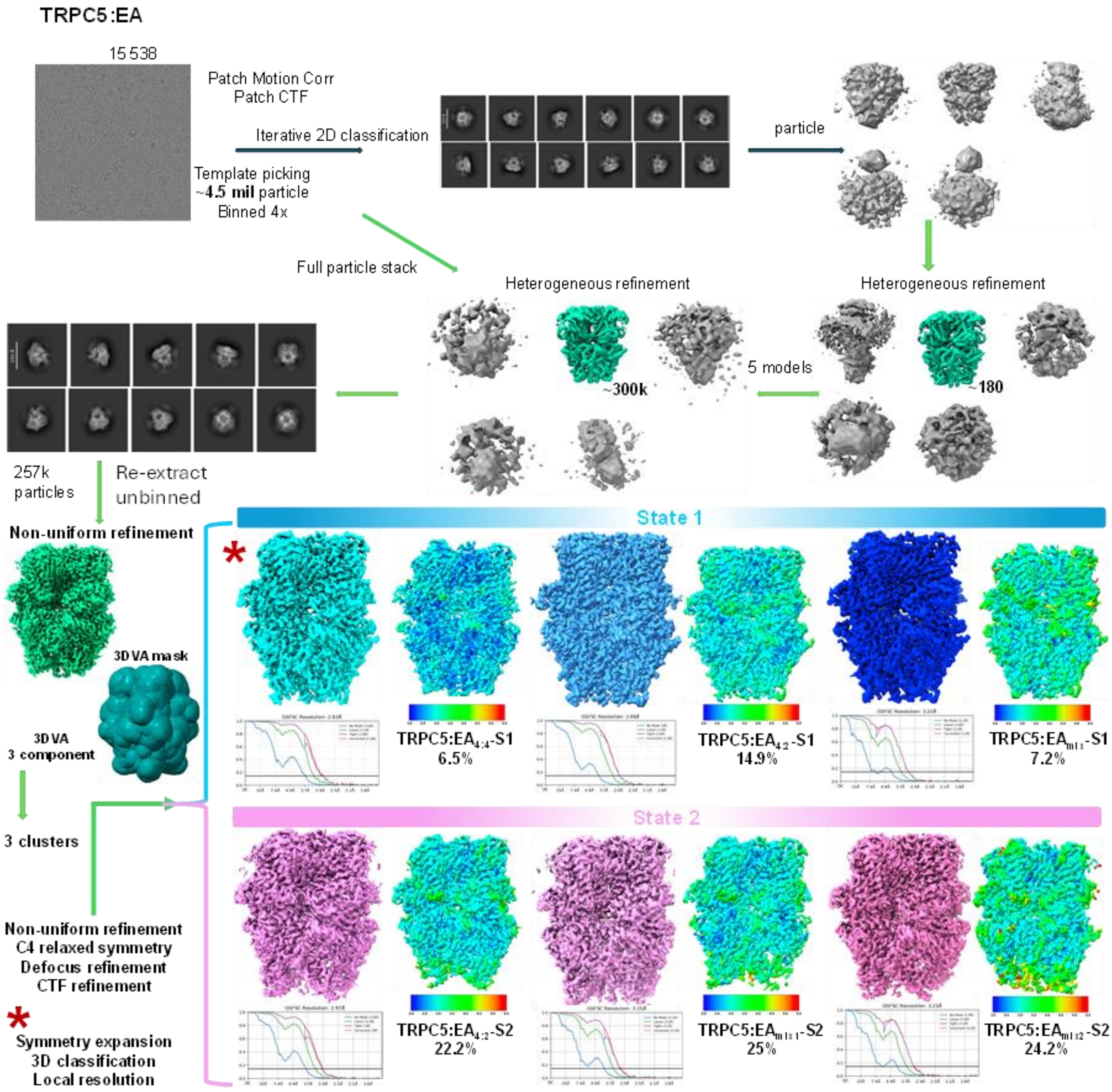

Supplementary Figure 8. Cryo-EM data processing workflow and map resolution of TRPC5:EA structures.

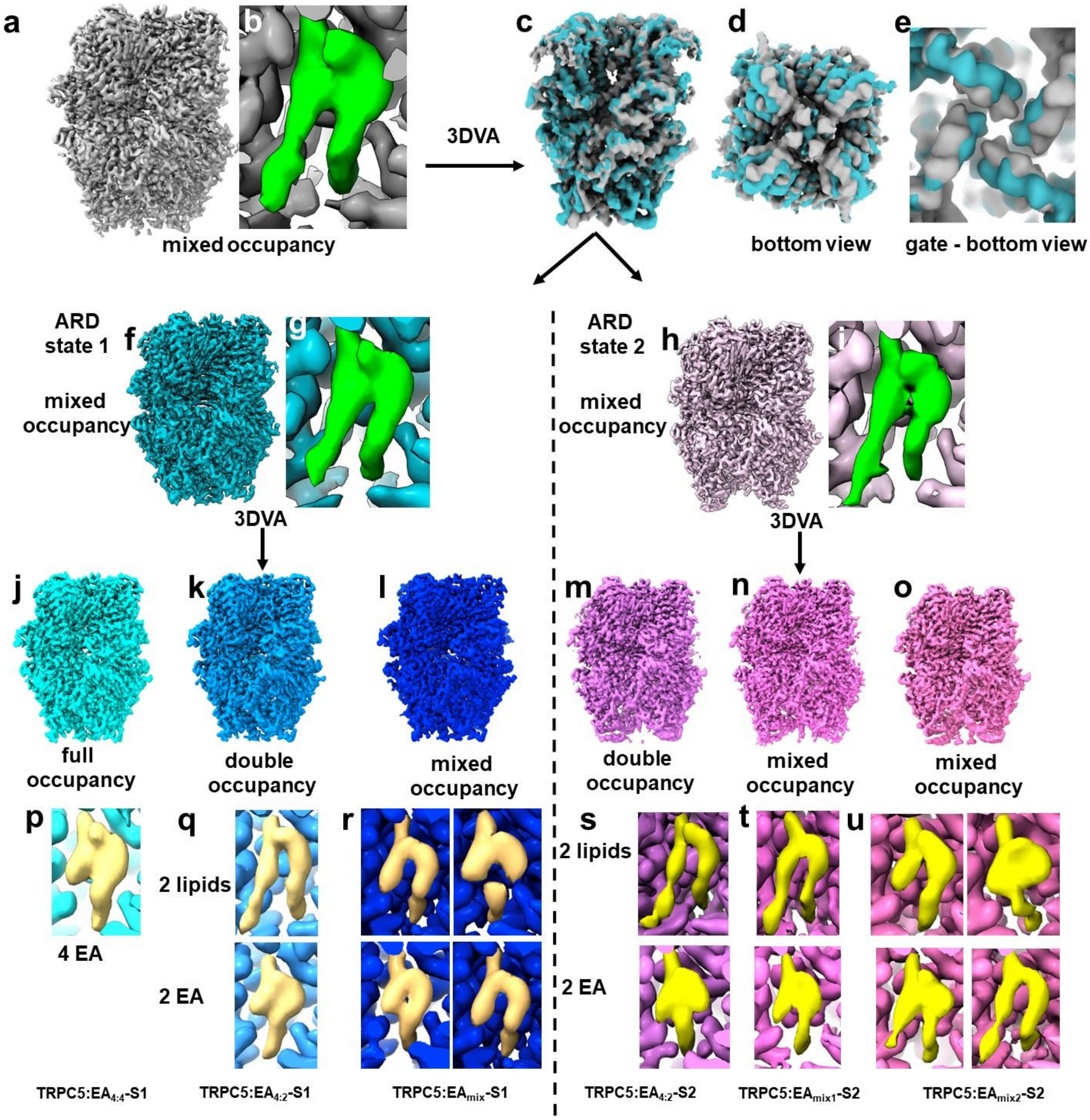

Supplementary Figure 9. Structures of TRPC5:EA reveal multiple EA/lipid binding stoichiometries. **a,b,** Initial cryo-EM map of TRPC5:EA (a), with a close-up of the EA binding site showing ambiguous density (green; b)**. c-e,** 3DVA analysis and separation of two ARD states of TRPC5 illustrated with a side view (c), bottom view (d) and a close-up on the lower gate (e). **f-i,** Cryo-EM maps of ARD state 1 (f) and ARD state 2 (h) and close-ups on the respective EA binding sites (g,i) showing ambiguous density (green). **j-l,** ARD state 1 cryo-EM maps with different EA/lipid binding stoichiometries (4:4 in cyan; 4:2 in light blue; mixed in dark blue). **m-o,** ARD state 2 cryo-EM maps with different EA/lipid binding stoichiometries (4:2 in magenta; mixed1 in light magenta; mixed2 in pink). **p-r,** Close-ups of EA binding sites of ARD state 1 maps showing different non protein densities (cream). **s-u,** Close-ups of EA binding sites of ARD state 2 maps showing different non protein densities (yellow).

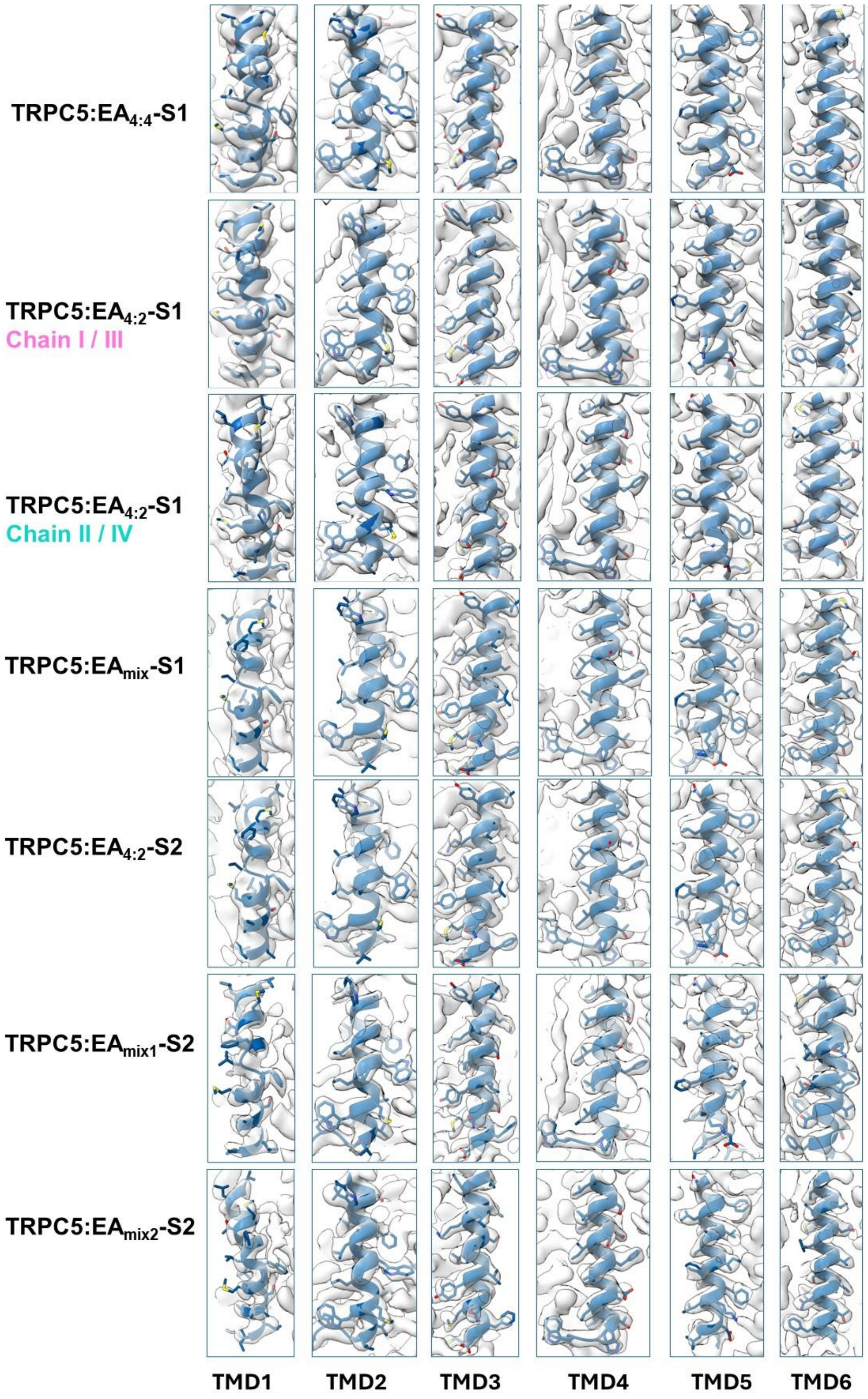

Supplementary Figure 10. **Data quality of TRPC5:EA structures illustrated by the fit of the six transmembrane domains (blue) in the EM maps (grey).**

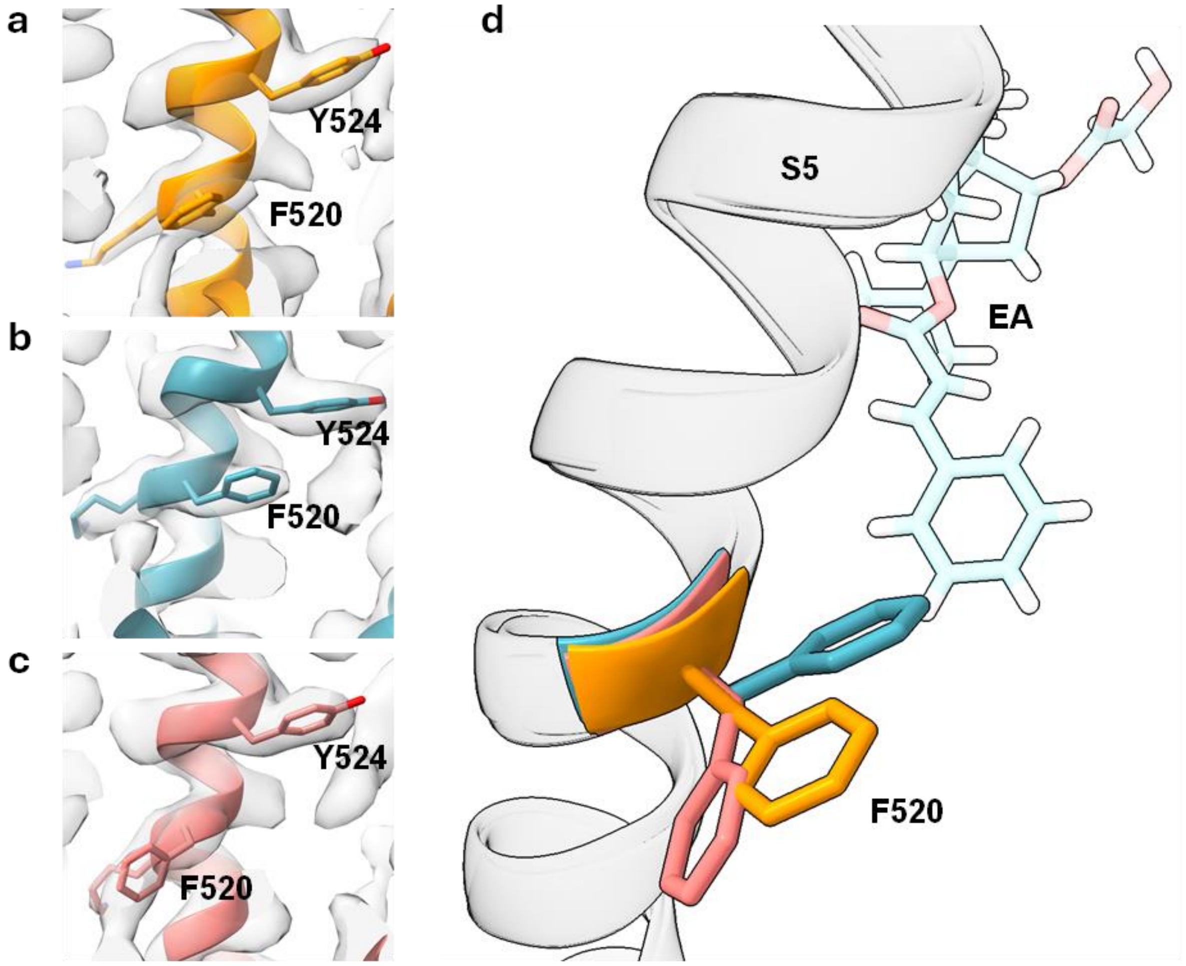

Supplementary Figure 11. Dynamics of Phe520 in TRPC5 activation. a, TRPC5^{PA}. **b,** TRPC5:EA^{PA}-S1. **c,** TRPC5:EA$_{4:4}$-S1. **d,** Superimposition of S5 helix from the three structures showing different orientation of Phe520.

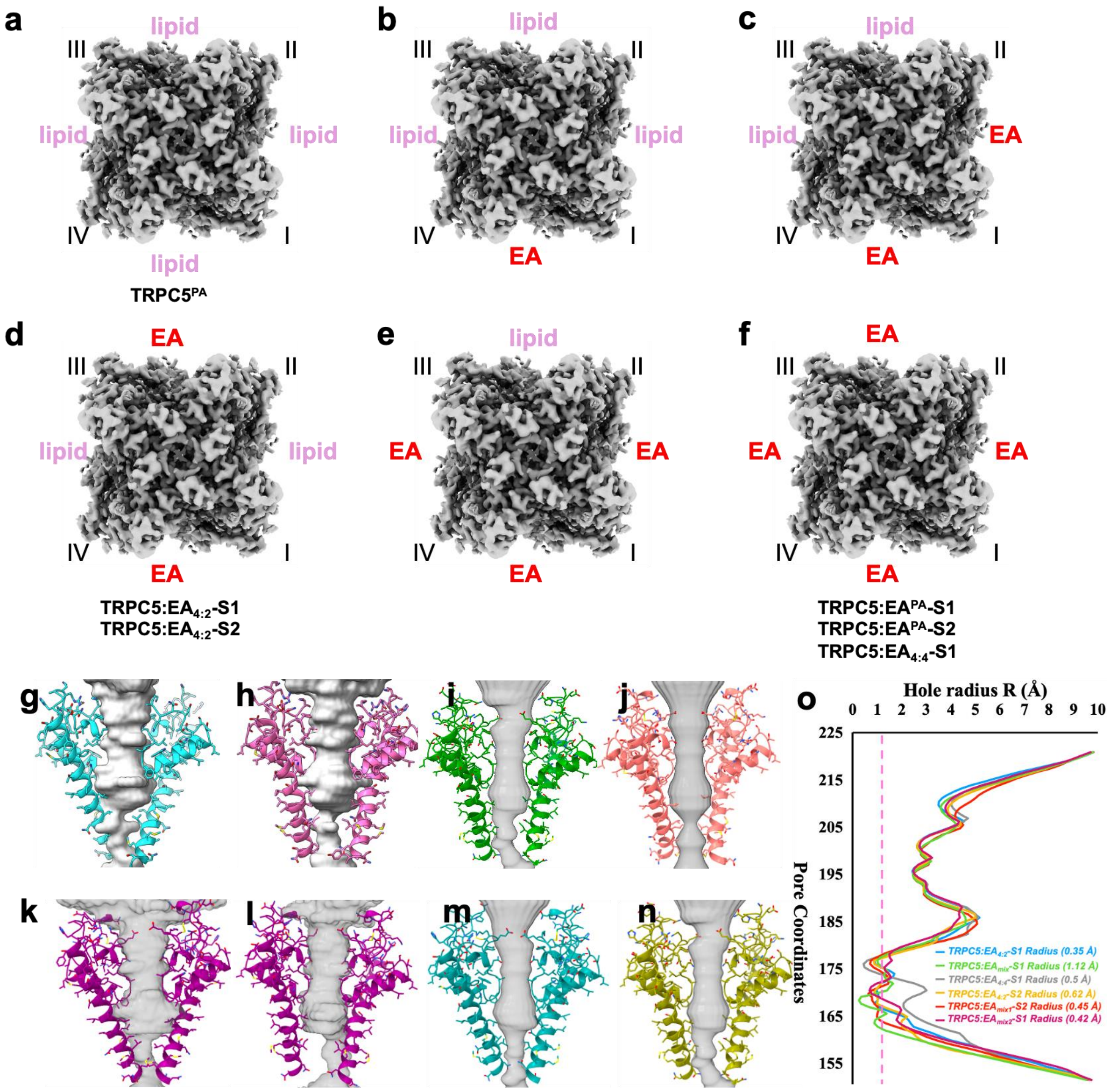

Supplementary Figure 12. Stoichiometry and pore analysis of TRPC5:EA structures. a-f, Possible stoichiometries of EA:lipid in TRPC5. Structures of three possible arrangements were determined unambiguously: $TRPC5^{PA}$ (a); TRPC5:$EA_{4:2}$-S1 and TRPC5:$EA_{4:2}$-S2 (d); TRPC5:EA^{PA}-S1, TRPC5:EA^{PA}-S2 and TRPC5:$EA_{4:4}$-S1 (f). Three additional structures (TRPC5:EA_{mix}-S1, TRPC5:EA_{mix1}-S2 and TRPC5:EA_{mix2}-S2 represent mixed populations of arrangements shown in panels (b-e) and could not be separated further. **g-h,** Pore shape analysis of TRPC5:$EA_{4:2}$-S1, with projections showing TRPC5 subunits I and III (cyan; g) and TRPC5 subunits II and IV (pink; h). **i,j,** Pore shape analysis of TRPC5:EA_{mix}-S1 (i), and TRPC5:$EA_{4:4}$-S1 (j). **k,l,** Pore shape analysis of TRPC5:$EA_{4:2}$-S2, with projections showing TRPC5 subunits I and III (k) and TRPC5 subunits II and IV (l). **m,n,** Pore shape analysis of TRPC5:EA_{mix1}-S2 (m) and TRPC5:EA_{mix2}-S2 (n). **o,** Plot of the pore radii of TRPC5 structures against pore coordinates down the channels. The pink dashed line indicates where the pore becomes too narrow for water to pass through (1.2 Å). Pore radius of TRPC5:EA structures. Pore shape analysis and calculation of pore radii was performed using PoreAnalyzer.

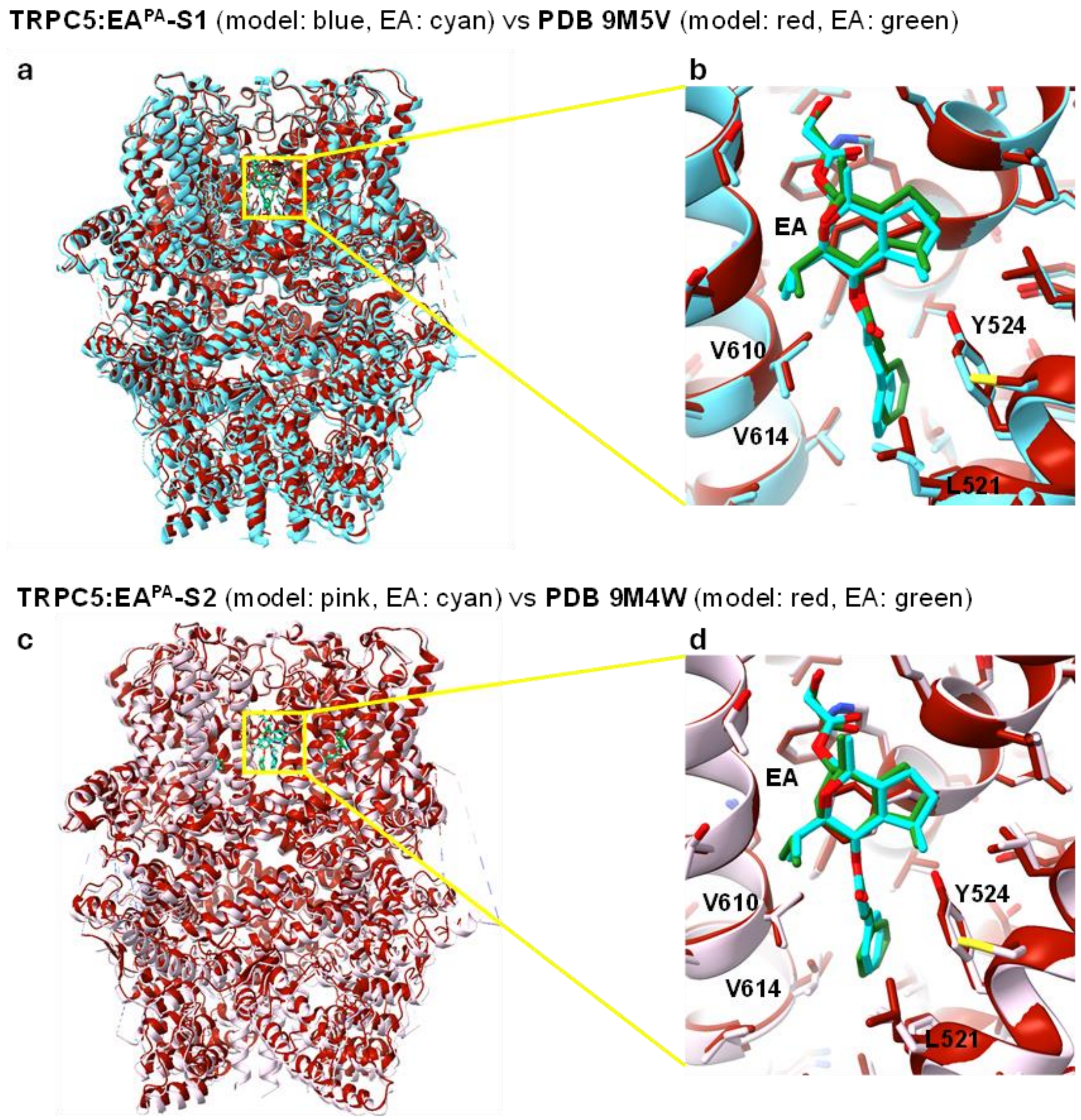

Supplementary Figure 13. Comparison of our TRPC5:EA^{PA} structures to recently reported TRPC5:EA structures by Chen et al.[63] **a**, Overlay of TRPC5:EA^{PA}-S1 (model: blue, EA: cyan) and PDB 9M5V (model: red, EA: green). Both models represent TRPC5 in state 1. **b**, Close up of the EA binding sites in (a). c, Overlay of TRPC5:EA^{PA}-S2 (model: blue, EA: cyan) and PDB 9M5V (model: red, EA: green). Both models represent TRPC5 in state 2. **d**, Close up of the EA binding site in (c).

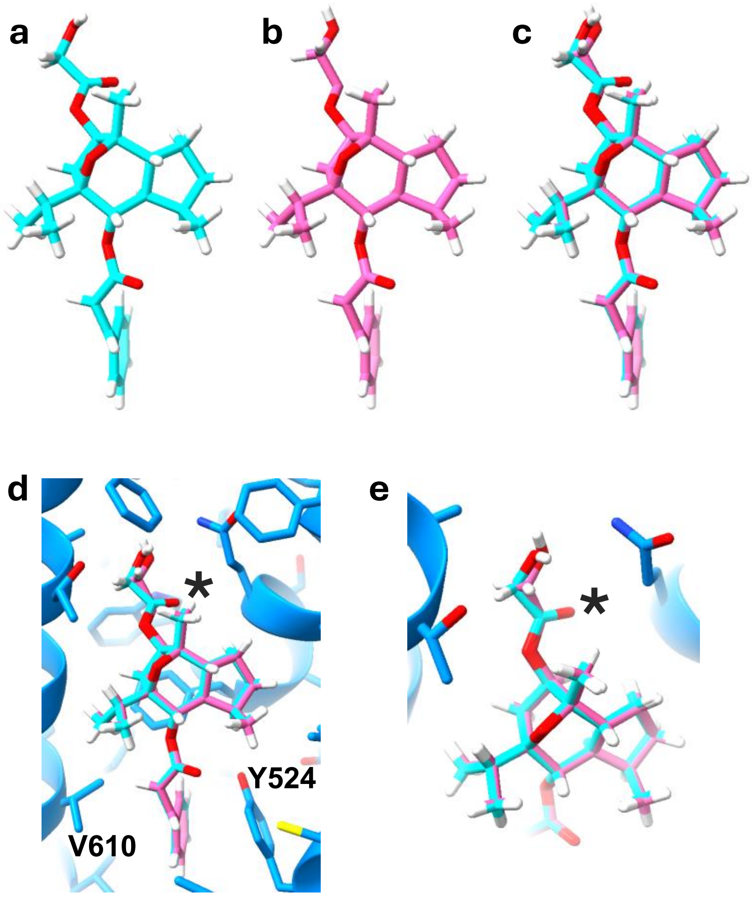

Supplementary Figure 14. Structural comparison of the natural product EA and its synthetic analogue A54. **a**, The TRPC5 agonist EA as bound in TRPC5:EA^{PA}-S1. **b**, The TRPC5 antagonist A54 as predicted to bind to TRPC5:EA^{PA}-S1 (based on molecular replacement of EA and real space refinement in Coot). **c**, overlay of EA (a) and A54 (b). **d,e,** Overlay of EA and A54 in the ligand binding site of the TRPC5:EA^{PA}-S1 structure. The chemical difference between the two compounds (ester carbonyl in the glycolate side chain of EA; ether CH2 group in A54) is indicated with an asterisk.

Experiment 1

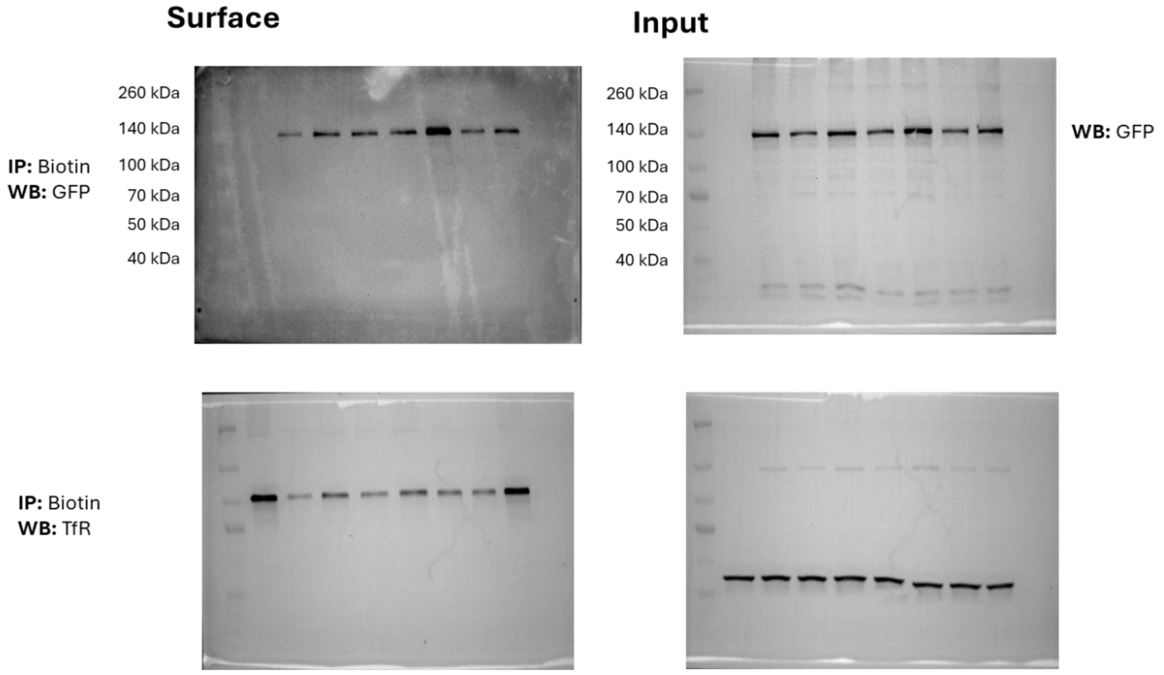

Experiment 2

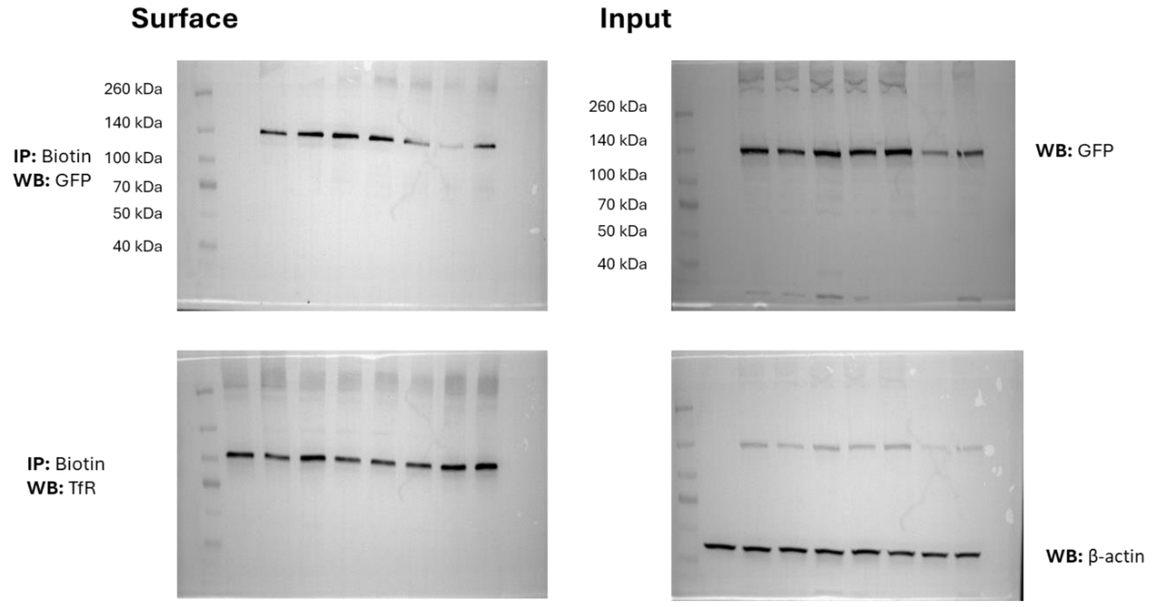

Experiment 3

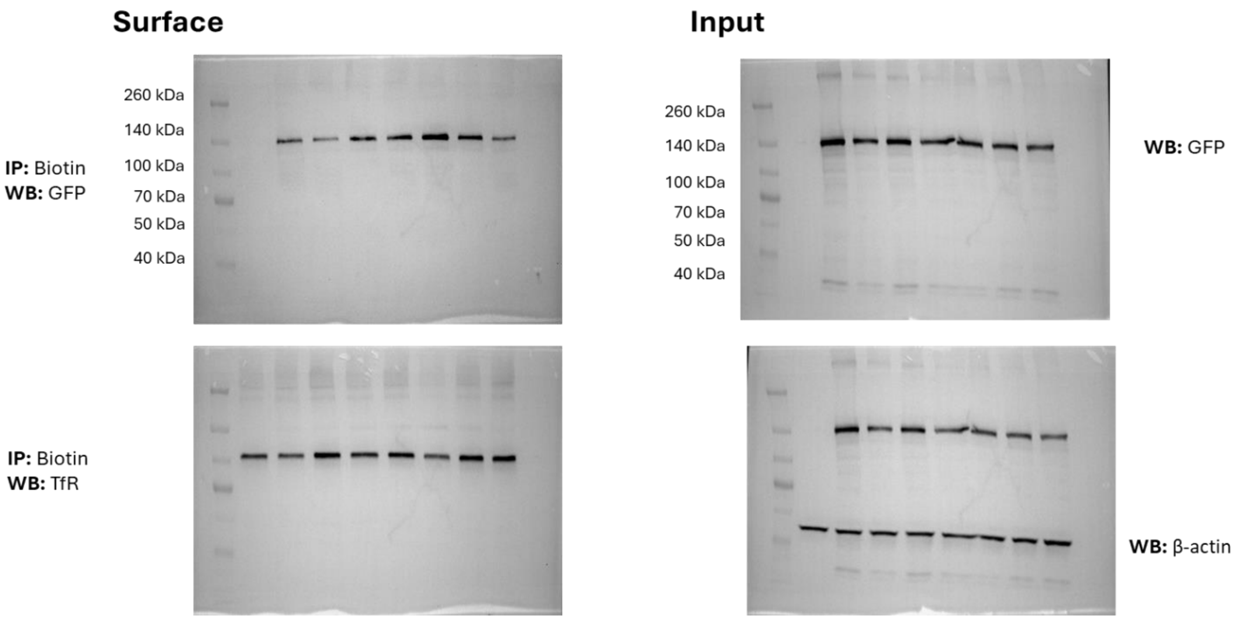

Supplementary Figure 15. Complete western blots for surface biotinylation experiments described and analysed in Supplementary Figure 5d (three independent experiments).

Supplementary Table 1. Cryo-EM data collection, refinement and validation statistics.

Structure<br>PDB<br>EMDB	**TRPC5^{PA}**<br>9RRF<br>EMD-54186	**TRPC5:EA^{PA}-S1**<br>9RRM<br>EMD-54187	**TRPC5:EA^{PA}-S2**<br>9RRN<br>EMD-54188	**TRPC5:EA$_{4:4}$-S1**<br>9RRU<br>EMD-54204	**TRPC5:EA$_{4:2}$-S1**<br>9RRQ<br>EMD-54193	**TRPC5:EA$_{mix}$**<br>9RRO<br>EMD-54189	**TRPC5:EA$_{4:2}$-S2**<br>9RVV<br>EMD-54291	**TRPC5:EA$_{mix1}$-S2**<br>9RSH<br>EMD-54219	**TRPC5:EA$_{mix2}$-S2**<br>9RSG<br>EMD-54218
**Data collection and processing**									
Magnification	165k	270k	270	165k	165k	165k	165k	165k	165k
Voltage (kV)	300	300	300	300	300	300	300	300	300
Electron exposure (e^{-}/Å^{2})	35.46	40.65	40.65	34.75	34.75	34.75	34.75	34.75	34.75
Defocus range (μm)	-0.7 to -3.0	-0.7 to -3.0	-0.7 to -3.0	-0.7 to -3.0	-0.7 to -3.0	-0.7 to -3.0	-0.7 to -3.0	-0.7 to -3.0	-0.7 to -3.0
Pixel size (Å)	0.74	0.46	0.46	0.82	0.82	0.82	0.82	0.82	0.82
Symmetry imposed	C4	C4	C4	C4	C2	C1	C1	C1	C1
Final particle images (no.)	167,808	37,807	69,865	15.953	38,365	18,655	57,236	63,619	62,384
Map resolution (Å)	2.4	2.5	2.5	2.8	2.9	3.2	3.0	3.1	3.2
FSC threshold	0.143	0.143	0.143	0.143	0.143	0.143	0.143	0.143	0.143
**Refinement**									
Initial model	ModelAngelo	ModelAngelo	ModelAngelo	ModelAngelo	ModelAngelo	ModelAngelo	ModelAngelo	ModelAngelo	ModelAngelo
Map sharpening *B* factor (Å^{2})	-77.7	-59.6	-71.4	-57.0	-48.5	-36.9	-57.2	-60.2	-56.4
Model composition									
Non-hydrogen atoms	23476	23732	23240	23572	23615	21562	20689	19608	20866
Protein residues	2720	2772	2716	2752	2749	2647	2454	2412	2563
Ligands	20	20	20	20	20		16		
Bonds (RMSD)									
Length (Å) (# > 4σ)	0.005(0)	0.003(0)	0.001(0)	0.003(0)	0.003(0)	0.003	0.003(0)	0.002(0)	0.002(0)
Angles (°) (# > 4σ)	0.461(0)	0.461(0)	0.343(0)	0.424(0)	0.463(2)	0.550(0)	0.383(0)	0.569(1)	0.537(0)
**Validation**									
**MolProbity score**	1.8	2.03	1.74	1.78	2	1.57	1.87	1.59	1.71
Clash score	13.94	16.87	17.36	17.37	20.72	5.57	20.4	6.0	7.79
Ramachandran plot (%)									
Outliers	0.0	0.00	0.15	0.15	0.0	0.0	0.0	0.0	0.24
Allowed	2.83	3.07	1.49	1.62	3.09	2.62	2.05	2.31	3.84
Favored	97.17	96.93	98.36	98.23	96.91	97.38	97.95	97.69	95.92
Rotamer outliers (%)	0.82	1.45	0.78	1.14	0.82	1.56	1.19	1.82	0.48
B factors									
Protein	3.54/171.3/69.3	9.6/179.4/80.5	5.24/171.9/73.8	21.7/162.1/79.7	41.7/193.7/100.7	37.9/180.7/98.6	25.7/183.9/94.4	32/189.6/97.8	9.17/184.2/63.0
Ligand	18.4/180.6/60.4	25.2/170.5/70.1	17.5/153.8/58.1	46.2/177.1/82.1	67.6/214.6/103.2		54.4/211.0/88.5		
**Model vs. Data**									
CC (mask)	0.91	0.82	0.8	0.88	0.89	0.85	0.86	0.85	0.87
CC (box)	0.71	0.64	0.64	0.62	0.6	0.56	0.7	0.7	0.7
CC (peaks)	0.74	0.61	0.61	0.65	0.6	0.56	0.65	0.69	0.65
CC (volume)	0.88	0.79	0.77	0.86	0.87	0.84	0.84	0.83	0.85
Mean CC for ligands	0.71	0.67	0.7	0.67	0.69		0.68		

Supplementary Table 2. Overview of TRPC5 residues that could not be modelled in TRPC5 structures.

Structure	Subunits			
	I	II	III	IV
**TRPC5^{PA}**	1-16, 120-133, 275-282, 667-703, 755-765			
**TRPC5:EA^{PA}-S1**	1-14, 120-134, 279-284, 666-699, 762-765			
**TRPC5:EA^{PA}-S2**	1-16, 120-133, 276-284, 666-699, 753-765			
**TRPC5:EA$_{4:2}$-S1**	1-15, 120-133, 276-284, 665-700, 763-765.	1-15, 120-133, 276-284, 665-700, 763-765.	1-15, 120-133, 276-284, 665-700, 763-765.	1-15, 119-134, 276-284, 665-700, 762-765.
**TRPC5:EA$_{4:4}$-S1**	1-14, 120-134, 276-284, 635, 666-702, 765.			
**TRPC5:EA$_{mix}$-S1**	1-15, 119-134, 274-285,388-392,423-430,631-633, 665-702, 764-765.	1-15, 119-134, 274-285, 388-392,423-430, 631-633, 665-703, 764-765.	1-15, 119-134, 274-285, 388-392,423-430, 631-633, 665-702, 764-765.	1-15, 119-134, 274-285, 388-392, 423-430, 630-633, 665-702, 764-765.
**TRPC5:EA$_{mix1}$-S2**	1-15, 43-69, 93-95, 119-133, 275-284, 387-391, 423-432, 666-700, 733-765.			
**TRPC5:EA$_{mix2}$-S2**	1-15, 119-133, 277-284, 386-390, 666-701, 747-765.	1-15, 119-134, 274-286, 386-390, 666-703, 749-765.	1-34, 119-134, 273-286, 388-393, 665-703, 733-765.	1-41, 118-135, 275-284, 388-397, 664-705, 734-765.
**TRPC5:EA$_{4:2}$-S2**	1-15,28-37, 41-64, 119-133, 275-284, 385-392, 666-700, 750-765.	1-15, 28-37, 41-64 119-133, 275-284, 385-392, 629-634, 666-700, 747-765.	1-15, 28-37, 41-64 119-133, 275-284, 385-392, 628-632, 666-700, 750-765.	1-15, 28-37, 41-64 119-133, 275-284, 385-392, 666-700, 750-765.

Supplementary Table 3. Root mean square deviations of the TRPC5 models. Global RMSD across all atom pairs in purple; global RMSD between pruned atom pairs in blue.

	$TRPC5^{PA}$	$TRPC5{:}EA^{PA}$-S1	$TRPC5{:}EA^{PA}$-S2	$TRPC5{:}EA_{4:2}$-S1	$TRPC5{:}EA_{4:4}$-S1	$TRPC5{:}EA_{mix}$-S1	$TRPC5{:}EA_{4:2}$-S2	$TRPC5{:}EA_{mix1}$-S2	$TRPC5{:}EA_{mix2}$-S2
$TRPC5^{PA}$		0.63 Å	0.53 Å	0.69 Å	0.59 Å	0.65 Å	0.52 Å	0.52 Å	0.6 Å
$TRPC5{:}EA^{PA}$-S1	1.4 Å		0.47 Å	0.66 Å	0.68 Å	0.65 Å	0.72 Å	0.64 Å	0.63 Å
$TRPC5{:}EA^{PA}$-S2	0.6 Å	1.5 Å		0.69 Å	0.71 Å	0.70 Å	0.64 Å	0.68 Å	0.55 Å
$TRPC5{:}EA_{4:2}$-S1	2.9 Å	1.6 Å	2.1 Å		0.44 Å	0.51 Å	0.76 Å	0.51 Å	0.69 Å
$TRPC5{:}EA_{4:4}$-S1	2.4 Å	0.9 Å	2.2 Å	1.6 Å		0.45 Å	0.63 Å	0.51 Å	0.59 Å
$TRPC5{:}EA_{mix}$-S1	2.2 Å	0.8 Å	2.2 Å	0.8 Å	0.7 Å		0.70 Å	0.57 Å	0.52 Å
$TRPC5{:}EA_{4:2}$-S2	1.7 Å	1.5 Å	1.2 Å	1.2 Å	1.6 Å	1.4 Å		0.55 Å	0.65 Å
$TRPC5{:}EA_{mix1}$-S2	2.08 Å	2.1 Å	1.6 Å	1.3 Å	1.2 Å	1.3 Å	2.1 Å		0.52 Å
$TRPC5{:}EA_{mix2}$-S2	2.6 Å	2.3 Å	2.1 Å	2.7 Å	2.4 Å	1.0 Å	0.7 Å	0.9 Å	
