# Supplementary material for: (-)-Englerin A binding to human TRPC5 exposes an aromatic interaction network in channel activation
Source: Nat Commun. 2026 Apr 29;17:5259. doi: 10.1038/s41467-026-71840-y (PMC13263324; doi:10.1038/s41467-026-71840-y)
Supplement: Supplementary file 2 — Description of Additional Supplementary Files [file 41467_2026_71840_MOESM2_ESM.pdf]

Description of Additional Supplementary Files

File name: Supplementary Movie 1
Description: TRPC5 structural dynamics. Our TRPC5 data set was analysed using 3DVA and the output was visualised using ‘simple mode’ in CryoSPARC. The resulting movie shows transitions between the ARD states of TRPC5 and the symmetry break in the channel pore.
