# Supplementary material for: (-)-Englerin A binding to human TRPC5 exposes an aromatic interaction network in channel activation
Source: Nat Commun. 2026 Apr 29;17:5259. doi: 10.1038/s41467-026-71840-y (PMC13263324; doi:10.1038/s41467-026-71840-y)
Supplement: Supplementary file 4 — Reporting Summary [file 41467_2026_71840_MOESM4_ESM.pdf]

nature portfolio

Corresponding author(s): Sebastian A. Porav; Robin S. Bon

Last updated by author(s): Feb 21, 2026

Reporting Summary

Nature Portfolio wishes to improve the reproducibility of the work that we publish. This form provides structure for consistency and transparency in reporting. For further information on Nature Portfolio policies, see our Editorial Policies and the Editorial Policy Checklist.

Statistics

For all statistical analyses, confirm that the following items are present in the figure legend, table legend, main text, or Methods section.

n/a	Confirmed	
☐	☒	The exact sample size (*n*) for each experimental group/condition, given as a discrete number and unit of measurement
☐	☒	A statement on whether measurements were taken from distinct samples or whether the same sample was measured repeatedly
☐	☒	The statistical test(s) used AND whether they are one- or two-sided<br>*Only common tests should be described solely by name; describe more complex techniques in the Methods section.*
☒	☐	A description of all covariates tested
☐	☒	A description of any assumptions or corrections, such as tests of normality and adjustment for multiple comparisons
☐	☒	A full description of the statistical parameters including central tendency (e.g. means) or other basic estimates (e.g. regression coefficient) AND variation (e.g. standard deviation) or associated estimates of uncertainty (e.g. confidence intervals)
☐	☒	For null hypothesis testing, the test statistic (e.g. *F*, *t*, *r*) with confidence intervals, effect sizes, degrees of freedom and *P* value noted<br>*Give P values as exact values whenever suitable.*
☒	☐	For Bayesian analysis, information on the choice of priors and Markov chain Monte Carlo settings
☒	☐	For hierarchical and complex designs, identification of the appropriate level for tests and full reporting of outcomes
☒	☐	Estimates of effect sizes (e.g. Cohen's *d*, Pearson's *r*), indicating how they were calculated

Our web collection on statistics for biologists contains articles on many of the points above.

Software and code

Policy information about availability of computer code

Data collection	Electrophysiology data were collected using an Axopatch 200B amplifier and the software pCLAMP 10.6 (Molecular Devices).<br>Data were filtered using the Digidata 1550B analog-to-digital converter controlled by Clampex 10.6 (Molecular Devices).<br>As related to TRPC5 structural biology: Cryo-EM data were collected using the standard EPU data collection software installed by FEI EPU 3.1
Data analysis	Electrophysiology data were analysed using Clampfit 10 and 11 (Molecular Devices), SigmaPlot 10 (Systat Software Inc.) and OriginPro 2021 (OriginLab Corporation).<br>As related to TRPC5 structural biology: Data were pre-processed (Motion correction and CTF estimation) in CryoSPARC v4.4 and v4.6 while particle picking, and data processing was done in Cryosparc v4.4 and v4.6. Initial models were generated using ModelAngelo, and manually adjusted in Coot (0.9.8.8) while for model refinement we used PHENIX (1.21rc1). Model validation, as reported in the paper, was conducted using PHENIX and Molprobity. UCSF ChimeraX (1.8) and PyMOL (v2.6 LTS) were used for visualisation and figure preparation. Pore shape and radius were calculated using a local installation of PoreAnalyser, helix curvature with HELANAL-Plus and Bendix inside VMD (v.1.9.4a57).<br>Arpeggio was used to identify aromatic interactions.

For manuscripts utilizing custom algorithms or software that are central to the research but not yet described in published literature, software must be made available to editors and reviewers. We strongly encourage code deposition in a community repository (e.g. GitHub). See the Nature Portfolio guidelines for submitting code & software for further information.

Data

Policy information about availability of data

All manuscripts must include a data availability statement. This statement should provide the following information, where applicable:

- Accession codes, unique identifiers, or web links for publicly available datasets
- A description of any restrictions on data availability
- For clinical datasets or third party data, please ensure that the statement adheres to our policy

Cryo-EM models and maps are available via the PDB and EMDB, respectively (TRPC5PA: 9RRF / EMD-54186, TRPC5:EAPA-S1: 9RRM / EMD-54187, TRPC5:EAPA-S2: 9RRN / EMD-54188, TRPC5:EA4:4-S1: 9RRU / EMD-54204, TRPC5:EA4:2-S1: 9RRQ / EMD-54193, TRPC5:EAmix-S1: 9RRO / EMD-54189, TRPC5:EA4:2-S2: 9RVV / EMD-54291, TRPC5:EAmix1-S2: 9RSH / EMD 54219, TRPC5:EAmix2-S2: 9RSG / EMD-54218). Raw data was deposited on EMPIAR under accession codes: EMPIAR-13164, EMPIAR-13160, EMPIAR-13162. Details of cryoEM structures are provided in Extended Data Table 1. Example data for intracellular calcium recordings, patch-clamp electrophysiology and surface biotinylation experiments are displayed in Figure 2, Figure 4, Figure 5 and Extended Data Figures 4-6. Full western blots for surface biotinylation experiments are provided in Supporting Figure 1. Other data and materials are available from the corresponding authors upon reasonable request.

Research involving human participants, their data, or biological material

Policy information about studies with human participants or human data. See also policy information about sex, gender (identity/presentation), and sexual orientation and race, ethnicity and racism.

Reporting on sex and gender	n/a
Reporting on race, ethnicity, or other socially relevant groupings	n/a
Population characteristics	n/a
Recruitment	n/a
Ethics oversight	n/a

Note that full information on the approval of the study protocol must also be provided in the manuscript.

Field-specific reporting

Please select the one below that is the best fit for your research. If you are not sure, read the appropriate sections before making your selection.

☒ Life sciences ☐ Behavioural & social sciences ☐ Ecological, evolutionary & environmental sciences

For a reference copy of the document with all sections, see nature.com/documents/nr-reporting-summary-flat.pdf

Life sciences study design

All studies must disclose on these points even when the disclosure is negative.

Sample size	n=3 for calcium recordings and surface biotinylation experiments<br>Electrophysiology: At least four independent electrophysiological recordings were performed for each experimental group.
Data exclusions	No data exclusions.
Replication	Data for calcium recordings and surface bioitinylation experiments from 3 separate transfections<br>Electrophysiology: Each experimental group was prepared using at least two independent transfections.
Randomization	Related to TRPC5 structural biology: N/A; no clinical samples or animal samples were used. Randomization was used in CryoSPARC to estimate the final resolution of cryo-EM maps.
Blinding	Related to TRPC5 structural biology. N/A all samples for TRPC5 cryo-EM analysis were prepared together and the experimenter was aware about the nature and particularities of the compounds.

Behavioural & social sciences study design

All studies must disclose on these points even when the disclosure is negative.

Study description	n/a
Research sample	n/a
Sampling strategy	n/a
Data collection	n/a
Timing	n/a
Data exclusions	n/a
Non-participation	n/a
Randomization	n/a

Ecological, evolutionary & environmental sciences study design

All studies must disclose on these points even when the disclosure is negative.

Study description	n/a
Research sample	n/a
Sampling strategy	n/a
Data collection	n/a
Timing and spatial scale	n/a
Data exclusions	n/a
Reproducibility	n/a
Randomization	n/a
Blinding	n/a

Did the study involve field work? ☐ Yes ☒ No

Field work, collection and transport

Field conditions	n/a
Location	n/a
Access & import/export	n/a
Disturbance	n/a

Reporting for specific materials, systems and methods

We require information from authors about some types of materials, experimental systems and methods used in many studies. Here, indicate whether each material, system or method listed is relevant to your study. If you are not sure if a list item applies to your research, read the appropriate section before selecting a response.

Materials & experimental systems

n/a	Involved in the study
☐	☒ Antibodies
☐	☒ Eukaryotic cell lines
☒	☐ Palaeontology and archaeology
☒	☐ Animals and other organisms
☒	☐ Clinical data
☒	☐ Dual use research of concern
☒	☐ Plants

Methods

n/a	Involved in the study
☒	☐ ChIP-seq
☒	☐ Flow cytometry
☒	☐ MRI-based neuroimaging

Antibodies

Antibodies used	GFP(Abcam; ab1218), beta-actin (ThermoFisher # MA1-140), TfR/CD71 (CST#13113)
Validation	*Describe the validation of each primary antibody for the species and application, noting any validation statements on the manufacturer's website, relevant citations, antibody profiles in online databases, or data provided in the manuscript.*

Eukaryotic cell lines

Policy information about cell lines and Sex and Gender in Research

Cell line source(s)	HEK293T cells (ATCC, CRL-3216), HEK293-F (ThermoFisher, R79007)
Authentication	No authentication of any cell line used in this study.
Mycoplasma contamination	cells were regularly tested for mycoplasma contamination (with negative results).
Commonly misidentified lines (See ICLAC register)	No commonly misidentified lines were used in this study.

Palaeontology and Archaeology

Specimen provenance	n/a
Specimen deposition	n/a
Dating methods	n/a

☐ Tick this box to confirm that the raw and calibrated dates are available in the paper or in Supplementary Information.

Ethics oversight	n/a

Note that full information on the approval of the study protocol must also be provided in the manuscript.

Animals and other research organisms

Policy information about studies involving animals; ARRIVE guidelines recommended for reporting animal research, and Sex and Gender in Research

Laboratory animals	n/a
Wild animals	n/a
Reporting on sex	n/a
Field-collected samples	n/a
Ethics oversight	n/a

Note that full information on the approval of the study protocol must also be provided in the manuscript.

Clinical data

Policy information about clinical studies

All manuscripts should comply with the ICMJE guidelines for publication of clinical research and a completed CONSORT checklist must be included with all submissions.

Clinical trial registration	n/a
Study protocol	n/a
Data collection	n/a
Outcomes	n/a

Dual use research of concern

Policy information about dual use research of concern

Hazards

Could the accidental, deliberate or reckless misuse of agents or technologies generated in the work, or the application of information presented in the manuscript, pose a threat to:

No	Yes	
☒	☐	Public health
☒	☐	National security
☒	☐	Crops and/or livestock
☒	☐	Ecosystems
☒	☐	Any other significant area

Experiments of concern

Does the work involve any of these experiments of concern:

No	Yes	
☒	☐	Demonstrate how to render a vaccine ineffective
☒	☐	Confer resistance to therapeutically useful antibiotics or antiviral agents
☒	☐	Enhance the virulence of a pathogen or render a nonpathogen virulent
☒	☐	Increase transmissibility of a pathogen
☒	☐	Alter the host range of a pathogen
☒	☐	Enable evasion of diagnostic/detection modalities
☒	☐	Enable the weaponization of a biological agent or toxin
☒	☐	Any other potentially harmful combination of experiments and agents

Plants

Seed stocks	n/a
Novel plant genotypes	n/a
Authentication	n/a

ChIP-seq

Data deposition

☐ Confirm that both raw and final processed data have been deposited in a public database such as GEO.

☐ Confirm that you have deposited or provided access to graph files (e.g. BED files) for the called peaks.

Data access links<br>*May remain private before publication.*	n/a
Files in database submission	n/a
Genome browser session<br>(e.g. UCSC)	n/a

Methodology

Replicates	n/a
Sequencing depth	n/a
Antibodies	n/a
Peak calling parameters	n/a
Data quality	n/a
Software	n/a

Flow Cytometry

Plots

Confirm that:

☐ The axis labels state the marker and fluorochrome used (e.g. CD4-FITC).

☐ The axis scales are clearly visible. Include numbers along axes only for bottom left plot of group (a 'group' is an analysis of identical markers).

☐ All plots are contour plots with outliers or pseudocolor plots.

☐ A numerical value for number of cells or percentage (with statistics) is provided.

Methodology

Sample preparation	n/a
Instrument	n/a
Software	n/a
Cell population abundance	n/a
Gating strategy	n/a

☐ Tick this box to confirm that a figure exemplifying the gating strategy is provided in the Supplementary Information.

Magnetic resonance imaging

Experimental design

Design type	n/a
Design specifications	n/a
Behavioral performance measures	n/a

Acquisition

Imaging type(s): n/a

Field strength: n/a

Sequence & imaging parameters: n/a

Area of acquisition: n/a

Diffusion MRI ☐ Used ☐ Not used

Preprocessing

Preprocessing software: n/a

Normalization: n/a

Normalization template: n/a

Noise and artifact removal: n/a

Volume censoring: n/a

Statistical modeling & inference

Model type and settings: n/a

Effect(s) tested: n/a

Specify type of analysis: ☐ Whole brain ☐ ROI-based ☐ Both

Statistic type for inference: n/a

(See Eklund et al. 2016)

Correction: n/a

Models & analysis

n/a	Involved in the study
☒	☐ Functional and/or effective connectivity
☒	☐ Graph analysis
☒	☐ Multivariate modeling or predictive analysis

Functional and/or effective connectivity: n/a

Graph analysis: n/a

Multivariate modeling and predictive analysis: n/a
