# Supplementary material for: (-)-Englerin A binding to human TRPC5 exposes an aromatic interaction network in channel activation
Source: Nat Commun. 2026 Apr 29;17:5259. doi: 10.1038/s41467-026-71840-y (PMC13263324; doi:10.1038/s41467-026-71840-y)
Supplement: Supplementary file 5 — Transparent Peer Review file [file 41467_2026_71840_MOESM5_ESM.pdf]

nature portfolio

Peer Review File

(-)-Englerin A binding to human TRPC5 exposes an aromatic interaction network in channel activation

Corresponding Author: Professor Robin Bon

This file contains all reviewer reports in order by version, followed by all author rebuttals in order by version.

Version 0:

Reviewer comments:

Reviewer #1

(Remarks to the Author)
In this study, Porov et al have determined the binding site for the TRPC4/TRPC5 agonist (-)-Englerin A (EA) on TRPC5 using cryoEM. They then performed functional experiments to both validate the binding site and assess the mechanisms of channel activation. While the authors do not observe the open state for TRPC5, that is known to be difficult to obtain for some ion channels and should not be an impediment to the publication of this work. The authors pick out several states of TRPC3 with different drug occupancies, which they interpret along with coordinating functional studies to understand the mechanisms by which EA may be able to open the channel. The identification of the binding mode for EA is impactful and the functional experiments presented corroborate the drug binding site nicely, but I think the authors need to more thoroughly sort their cryoEM data for the kind of multi-state analysis they perform in the second part of the paper.

Major issues:
-The authors planned their cryoEM experimental conditions well and obtained high quality datasets, which I would agree unambiguously identify the EA binding site. However, since the authors then go on in their work to interpret different states they observe in their heterogeneous data, they need to more cleanly separate out the mixtures of EA and lipid. The authors seem to have primarily used the larger conformational changes of the ARDs/CCD to guide particle sorting, resulting in every EA-bound structure having some degree of mixed lipid/EA occupancy. The authors have chosen to focus in the manuscript only on conformational changes at the pore and do not relate those changes to what they see happening at the ARDs/CCD, so it is unclear to me why they feel that this mode of sorting gives them the most useful results rather than focused classification only of the EA/lipid/S6 region. If the authors think the correlation between EA occupancy, S6 conformation, and the rotation of the ARDs/CCD is meaningful, they should discuss that in the text.
-I have concerns about the cryoEM data processing as well as model building, as detailed below.
Data Processing:
-For the two datasets with heterogeneity (EA/PA and EA only), the authors seem to have exclusively used the cryoSPARC 3D variability tool to sort conformational states using the entire molecule (perhaps they could show masks used for this technique in their workflow figures). While this type of analysis is useful for determining the overall movement in a dataset and can be used to effectively sort low resolution conformation differences, like the movements of entire domains, it can be much trickier to effectively use for sorting high resolution details, like drug occupancy and whether the S6 helix is alpha helical or has a pi bulge. The authors even say themselves that they see evidence for a mixed population of alpha helix and pi-bulge in both their EA/PA structures. I highly recommend that the authors use focused classification, whether in cryoSPARC or Relion, ideally with symmetry expansion, to carefully sort out occupancy for drug vs lipid and the helical status of S6 to get a clearer picture of their states. The authors could consider a similar approach to sorting as used in doi: 10.1038/s41594-024-01299-2, where focused classification could neatly sort very similar features.
-I also encourage the authors to carefully consider symmetry in the models where it is applied. There is clear indication of multiple conformations at the pi-bulge in both their EA/PA structures, but also at the bottom of S6 in their EA 4:4 structure. This may be due to heterogeneity that can be sorted out, but it could also be due to C4 averaging of particles that have subtle asymmetry.
Model Building:
-The authors have relied on Model Angelo to build their initial models; Model Angelo can be a good starting point, but it is not an infallible tool. The authors seem to have trusted it to build tricky/heterogeneous portions of the model rather than using better data processing approaches to resolve those areas of heterogeneity. For example, the bottom of the S6 helix is pretty messy in many of their structures, but frequently has residues modelled in despite that uncertainty. Additionally, there

are some areas without heterogeneity that are obviously incorrect, such as the 400-405 loop in the PA only model, that could be easily fixed manually.
-There are a couple of places in the paper where the authors discuss a novel local conformation they observe in their models but that region of the model is either not the best interpretation of the density (such as the register of the bulging loop at the base of S6 in the chain D of the EA 4:2 S1 structure) or one of a couple different conformations that could fit in clearly heterogeneous density (such as the bottom of S6 in the EA 4:4 structure). If the authors want to make a story about a novel conformation they observe in their data, they need to first make sure the region is not heterogeneous (or sort further if it is) and then fit the model with great care.

Minor Issues:
-Many of the structural figures were difficult to interpret or sometimes even mis-represented the actual structures. I found that I needed to go to the PDB structures to figure out where the residues/binding pockets the authors discussed were rather than being able to use the provided figures.
--Examples:
---Figure 4a-c (and subsequent figures of a similar style) were particularly difficult to interpret as the clipping from the depth cuing cut off the helix that F520 is attached to. Additionally, the authors do not label the helices (eg, S5, S6) in many of their figures or mention in the figure legends which helices are depicted in the figures.
---The helix-as-tube representation created odd representational artifacts in figures 5e and 7f that midrepresent the actual conformational changes the authors observed. So Figure 7f, for example, shows a complete dislocation of the the ends of two helical segments, when a coil representation shows that the actual conformational change is a modest bend caused by a pi bulge (compare 7f to 7p). I think the authors would be far better served by competely skipping the helix-as-tube representation and make backbone comparisons with a simple coil or ribbon representation.
---Since the authors are presenting F520 as a key residue for EA signal transduction, it would be very helpful if the authors could make a figure clearly comparing the three conformations for it they observe: Apo/PA, EA/PA, and the third conformation oriented out of the pore.
-The model provided for the PA only map is very slightly offset from the map - double check this in what you have deposited to the RCSB
-The pore profiles in extended data figure 11o don't seem to quite match up with the pores depicted. Specifically, the blue and yellow lines (corresponding to the two 4:2 conformations) are show as having very narrow selectivity filters, narrower than the key constriction diameter preventing the passage of water, while the other structures have much looser selectivity filters. Examination of the panels g,h,k and l, which show the 3D shape of the pore, are not consistent with that measurement.

Reviewer #2

(Remarks to the Author)
Review for NC 626931
In this report, Porav and colleagues describe several (eight) new cryo-EM structures of human TRPC5 bound by its agonist Englerin A (EA). Although multiple high-resolution TRPC5 structures, including several ligand-bound ones, have been made available, the EA-bound TRPC4 or TRPC5 structures have been proven to be difficult to obtain. Here, the authors tried two new strategies to preserve EA binding to TRPC5 during the sample preparation and succeeded in resolving the EA-bound structures, which have been long overdue, at excellent high resolutions (2.4-3.2 Å). Although they were unable to obtain an open structure for TRPC5 channels, the resolved intermediate and/or desensitized states are valuable for the understanding of TRPC5 structural dynamics and gating mechanisms, as well as the rationale design of drugs targeting TRPC5 and related channels. This work is significant because TRPC channels are strongly implicated in many physiological functions and diseases.
The cryo-EM structures are of high quality, and functional studies are thorough and informative. The following suggestions may help improve the manuscript.
1) The title of the paper seems to suggest that a Met-aromatic motif plays a critical role in TRPC5 channel activation. However, this point is not clearly presented in the Results section. Although S/π interactions are mentioned in several places, which particular S/π interaction is considered to be critical for channel activation is not disclosed. I suggest that the authors dedicate a specific section to present the data on this Met-aromatic motif and its function. Otherwise, the title should be modified to tone down the emphasis on this motif.
2) Pluronic acid (PA) was used to prepare EA-bound TRPC5. Does PA change the apparent affinity and/or efficacy of EA on TRPC5 channels?
3) In many TRP channel structures, the linker that connects CCD to the connecting helix crosses over the CCD of the neighbouring protomer so that its CCD interfaces with the ARD of the adjacent protomer. Is this also the case for TRPC5:EA^{PA}-S1 and TRPC5:EA^{PA}-S2, or only TRPC5:EA^{PA}-S1 but not TRPC5:EA^{PA}-S2?
4) What would be the possible reason for TRPC5$_{F520Y}$ being able to be activated by EA, but not by AM237, BTD, high extracellular Ca^{2+}, and carbachol in combination with Gd^{3+}? What would be the possible reason for TRPC5$_{Y524F}$ and TRPC5$_{Y524W}$ to selectively lose voltage-dependent activation (Figure 4d,e)? Since Tyr is present at this position only in TRPC4 and C5, but not in other EA-insensitive TRPCs, one would think that mutations at this site should mainly affect the activation by EA.
5) The I-V curve for TRPC5$_{L613A}$ appears linear. Can this be explained based on resolved new structures? As the authors pointed out in the discussion, a previous study has determined Asp633 to be the determinant of the double rectification I-V of TRPC5. Does the L613A substitution change the position of Asp633 in the channel structure?
6) Linn 4, a reference is needed for galangin modulation of TRPC5.

7) Lines 136-137 state that “The TRPC5^{PA} map displays a well-defined, characteristic lipid U-shape (Figure 1i)”. Please comment whether the PA treatment is required for seeing this lipid U-shape.
8) Lines 206-207, “This result suggests that leucine's bulky, hydrophobic side chain is important for the interaction between TRPC5 and EA”. Are you referring to a specific interaction discussed already or the interaction between TRPC5 and EA in a general sense? If for the latter, then are the side chains of Phe, Tyr, Met, Val, etc. discussed in this manuscript less important than that of Leu521? If not, why should Leu521 be specifically emphasized?
9) Lines 207-208, “TRPC5$_{L521F}$ was constitutively active at negative potentials (Extended Data Figure 4e)”. But based on the example shown, it looks like this mutant was constitutively active at both negative and positive potentials. Or does the basal activity represent leak currents?
10) Line 272, what “engages in an S/π interaction with Met513? Tyr628, Met623, or the C/π interaction between them?
11) Line 342-343, values for the pore radii should be given for TRPC5:EA^{PA}-S1 and TRPC5:EA^{PA}-S2.
12) Line 394 says “In TRPC5:EA$_{4:4}$-S1, the benzyl group of Phe520 is rotated by ~125° compared to TRPC5:EA^{PA}-S1”. How does it compare to TRPC5^{PA}?
13) Line 469, the second “TRPC5:EA$_{4:2}$-S1” should be “TRPC5:EA$_{4:2}$-S2”.
14) Line 486, “Leu506” should be “Ile506”.
15) Line 500, “The disappearance of the S6 π-bulge of subunits I/III...” Should this be “The disappearance of the S6 π-bulge of subunits II/IV…” based on that stated in lines 497-498? Likewise, in line 501, “the positional shift in subunits II/IV…” should be “the positional shift in subunits I/III…”.
16) Line 618, “voltage-current relationship” is more commonly expressed as “current-voltage relationship”.
17) In Figure 7b and c, should the pick helices in these two panels be the same helix at the same position? Then why are the number labels different between them? Also, should L618 in panel c actually be L617?
18) In Extended Data Figure 6g-j, for ease of reading, the color and style of the symbols should be kept consistent for the same drug concentration in these four panels.

Reviewer #3

(Remarks to the Author)
The manuscript by Sebastian A. Porav et al. reports a series of high-resolution cryo-EM structures of human TRPC5 in various functional states upon binding to the natural product agonist (-)-englerin A (EA). By integrating structural, biochemical, and mutagenesis approaches, the authors elucidate the EA binding site, identify key functional residues, and propose mechanistic insights into EA-mediated TRPC5 activation. This study offers important contributions to our understanding of TRPC channel regulation and provides a structural framework for rational drug design targeting the TRPC1/4/5 subfamily. While the study is of high interest and presents solid experimental data, several key issues require clarification or further elaboration to strengthen the manuscript’s impact and rigor.

Major Comments
1.Chen et al. (DOI: 10.1002/pro.70218) have recently reported cryo-EM structures of TRPC5 bound to EA. The authors should cite this work and provide a side-by-side comparison of key structural features. It is essential to clearly articulate the novel aspects and technical advantages of the current study relative to that prior work.
2.The authors report multiple EA-bound and apo structures, such as TRPC5:EAPA-S1, TRPC5:EAPA-S2, and TRPC5PA. Although the overall architecture appears similar, the manuscript should provide pairwise RMSD values between these conformations to quantify structural differences.
3.Figures 3a and 3b detail residue-level conformational shifts around the EA binding pocket. However, the manuscript lacks a comprehensive view of global pocket dynamics. The authors are encouraged to present comparative structural analyses (apo vs. EA-bound) of the full EA binding pocket before focusing on local residue movements. This will significantly improve mechanistic clarity.
4.Line 342 states that the pore radius “doubles” or “triples” in EA-bound structures. However, all presented conformations appear to correspond to closed states. The authors should: (1)Clarify how pore radius measurements were obtained. (2)Explicitly acknowledge that the captured conformations are non-conductive.(3)Discuss the possibility of using molecular dynamics simulations to explore transition pathways toward open states starting from EA-bound conformations.
5.At Line 83, the authors note that incubation with EA alone yielded apo structures, prompting the addition of pluronic acid (PA). Please specify the final PA concentration used. Also, clarify how the “TRPC5:EA structures in the absence of PA” (Line 367) were obtained. Were alternative solubilization strategies employed?
6.Although structurally similar to EA, the analogue A54 does not activate TRPC channels. The authors should provide structural or mechanistic insights to explain this functional divergence, ideally using structural overlays or binding pocket modeling.

Minor Comments
Figure 2a–c: Show all interactions within 5 Å. Hydrogen bonds should be depicted with dashed lines rather than solid lines, and this convention should be applied consistently throughout all figures.
Figure 2e: Maintain consistent axis scales with related panels to clearly highlight the functional loss of Y524A.
Figures 2d–j: Add a space in “EA (30nM)” for clarity.
Figure 3 vs. Figure 4: Ensure uniform schematic style and illustration conventions across all figures.
Line 345: Consider modeling alternate conformations for Asn626 as suggested by observed density.
Extended Data Figure 1: Color labels for TRPC5:EAPA-S1 and TRPC5:EAPA-S2 are reversed.
Abbreviations: Please add "SBS" to the abbreviations list.

Version 1:

Reviewer comments:

Reviewer #1

(Remarks to the Author)
In this revised manuscript, Porov et al have implemented reviewer recommendations to improve the interpretability and broaden the perspective of their work. My main comment during the first round of review was to request that the authors perform more exhaustive sorting of the heterogeneous regions of their cryoEM data. I understand that not every dataset cooperates and I appreciate that the authors put effort into this, resulting in two improved maps/models, and explained their process. I maintain my earlier view that this work is high quality and worth publishing, especially with the improvements made after the first round of review. However, there were some important oversights in the assembly of the current submission and I must remind the authors to be very meticulous in updating/assembling the manuscript.

1. The authors need to update the structures/maps in their figures where those structures/maps have changed since the initial submission.
a. This is particularly obvious in Figure 7, where the only change to the figure since the previous version is switching the helix-as-tube representation to a ribbon representation in 7e-f,i-j. However, the TRPC5:EA 4:2-S1 structure has been changed since the previous version, going from a C1 relaxed symmetry refinement to a C2 refinement. This structure now has a different and higher quality map, as well as a model which has changed due to refinement into that map. All panels of this figure must be updated to reflect the final maps and models. Since the authors are no longer comparing the two best chains of an asymmetric object, they should also now compare chains I and II rather than II and III.
b. The authors did further processing to improve the maps for both the TRPC5:EA 4:4-S1 and TRPC5:EA 4:2-S1 structures, but none of the main figures showing maps for these structures appear to have been updated from the previous version of the manuscript. While I have emphasized Figure 7 above, all figures using the maps/models for these structures need to be updated to match the RCSB/EMDB deposited structures. This includes Fig 5b, all of Fig 6, Extended Data Figure 10 and Extended Data Figure 12j-l, but the authors themselves should also carefully review all figures to ensure that they are presenting the correct versions of their structures.

2. In my initial review, I asked the authors about sorting their structures based on the ARD/CCD conformation. The explanation they gave in the response to reviewers was fine, but they made no alteration to explain this point in the text. The authors should add a sentence explaining that the two conformations are not expected to have different EA occupancy or be a result of EA binding and why they chose to use the ARD/CCD conformation to guide state separation.

3. In my initial review, I recommended that the authors remove all helix-as-tube representations in their figures as they inaccurately exaggerated the differences between structures. While the authors did fix this for Figure 7, they did not fix this in Figure 5e. The authors need to update this figure to remove the helix-as-tube representation.

4. I appreciate that the authors trimmed their structures to be a little more conservative about interpreting challenging density. While the authors say in the response to reviewers that they updated Extended Data Table 2 to reflect their altered structures, the table is identical to the table from the previous manuscript before these structures had been trimmed. I can see from the residue count in the updated Extended Data Table 1 that there should be quite a few differences.

Reviewer #2

(Remarks to the Author)
The revision has addressed the comments raised. The paper is much improved.

Reviewer #3

(Remarks to the Author)
The authors have nicely addressed the reviewer's concerns with new experiments. The new data are consistent with their conclusion， thus I recommend acceptance.

Version 2:

Reviewer comments:

Reviewer #1

(Remarks to the Author)
The authors have fully addressed my comments, the manuscript looks good to me.

Open Access This Peer Review File is licensed under a Creative Commons Attribution 4.0 International License, which permits use, sharing, adaptation, distribution and reproduction in any medium or format, as long as you give appropriate credit to the original author(s) and the source, provide a link to the Creative Commons license, and indicate if changes were made.
In cases where reviewers are anonymous, credit should be given to 'Anonymous Referee' and the source.
The images or other third party material in this Peer Review File are included in the article's Creative Commons license, unless indicated otherwise in a credit line to the material. If material is not included in the article's Creative Commons license and your intended use is not permitted by statutory regulation or exceeds the permitted use, you will need to obtain permission directly from the copyright holder.
To view a copy of this license, visit https://creativecommons.org/licenses/by/4.0/

Point-by-point response to editor's and reviewer's comments

We thank the editor and reviewers for their thorough evaluation of our study and their constructive comments, which have allowed us to improve the clarity and presentation of our work. We have made every effort to respond positively to all suggestions.

Corrections have been made where needed, and we have included new text, figures and references to provide additional clarification of our work and interpretation. We have included point-by-point responses to all comments (including quoted text and copies of new figures for any major changes) and provide a highlighted document to show all changes made to the manuscript.

Reviewer 1

In this study, Porov et al have determined the binding site for the TRPC4/TRPC5 agonist (-)-Englerin A (EA) on TRPC5 using cryoEM. They then performed functional experiments to both validate the binding site and assess the mechanisms of channel activation. While the authors do not observe the open state for TRPC5, that is known to be difficult to obtain for some ion channels and should not be an impediment to the publication of this work. The authors pick out several states of TRPC3 with different drug occupancies, which they interpret along with coordinating functional studies to understand the mechanisms by which EA may be able to open the channel. The identification of the binding mode for EA is impactful and the functional experiments presented corroborate the drug binding site nicely, but I think the authors need to more thoroughly sort their cryoEM data for the kind of multi-state analysis they perform in the second part of the paper.

We thank the reviewer for their detailed assessment of our manuscript, for their constructive suggestions regarding data processing and figure layout, and for catching errors.

Major issues:

1. The authors planned their cryoEM experimental conditions well and obtained high quality datasets, which I would agree unambiguously identify the EA binding site. However, since the authors then go on in their work to interpret different states they observe in their heterogeneous data, they need to more cleanly separate out the mixtures of EA and lipid. The authors seem to have primarily used the larger conformational changes of the ARDs/CCD to guide particle sorting, resulting in every EA-bound structure having some degree of mixed lipid/EA occupancy. The authors have chosen to focus in the manuscript only on conformational changes at the pore and do not relate those changes to what they see happening at the ARDs/CCD, so it is unclear to me why they feel that this mode of sorting gives them the most useful results rather than focused classification only of the EA/lipid/S6 region. If the authors think the correlation between EA occupancy, S6 conformation, and the rotation of the ARDs/CCD is meaningful, they should discuss that in the text.

Thank you for this question. We do not consider conformational changes of the ARDs/CCDs to be linked to EA binding. Indeed, the interactions between EA and TRPC5 residues are near-identical in both states (cf. **Figure 2a** vs **Figure 2b**), with the only difference being the nature of the dynamic hydrogen bond network made by EA's glycolate OH group. The latter is

reminiscent of the dynamic nature of the H-bond network of the OH group of Pico145's 3-hydroxypropyl substituent in TRPC5 – see Wright et al. 2020 (https://pubmed.ncbi.nlm.nih.gov/33230284/).

The different ARD/CCD conformations of TRPC5 we found were previously described in Won et al. 2023 (https://doi.org/10.1038/s41467-023-38281-3), who reported that this conformational change was not linked to binding of the $G\alpha_i$ subunit (a TRPC5 agonist) but present in different structures (both $G\alpha_i$-bound and 'apo'). In addition, while our original manuscript was under review, Chen et al. (2025) (https://doi.org/10.1002/pro.70218) reported structures of TRPC5:EA that are similar to our TRPC5:EA^{PA} structures, and suggested that the switching between ARDs/CCD states may be linked to Ca^{2+} concentration. We also have multiple (unpublished) data sets (apo and with various different bound agonists and antagonists) where we see this type of conformational change of the ARDs/CCD. We suspect that the free energy difference between the two states is very small and that the relative abundance of these states in cryoEM datasets is affected by subtle changes in the protein environment, including the reagents used for protein extraction and stabilisation (detergents/lipids, amphipol, nanodisc etc.).

During the image processing workflow, we have tested different strategies to sort the heterogeneity in our data, including 3D classification, with and without symmetry expansion, focused classification, and heterogenous refinement with different low-pass filtering and resolution limits. In our initial 3D classification (not shown), we focused on sorting the particles based on the presence or absence of EA using different masks. However, all our classes displayed similar EA density and poorly resolved ARDs/CCD. Upon symmetrically expanding the full particle stack and masking one monomer, we achieved a similar separation of the two states, but all classes still exhibited EA density at the binding sites. Additionally, in both states, we encountered several classes that continued to show relatively poor resolvability of the ARDs/CCD. When we applied 3D Variability Analysis (3DVA), we obtained a clearer separation and improved the quality of the stacks for the two states. We found that it was easier to first separate the 2 states and proceed with stoichiometry sorting, rather than other way around.

2. I have concerns about the cryoEM data processing as well as model building, as detailed below.

   Data Processing:

   a. For the two datasets with heterogeneity (EA/PA and EA only), the authors seem to have exclusively used the cryoSPARC 3D variability tool to sort conformational states using the entire molecule (perhaps they could show masks used for this technique in their workflow figures). While this type of analysis is useful for determining the overall movement in a dataset and can be used to effectively sort low resolution conformation differences, like the movements of entire domains, it can be much trickier to effectively use for sorting high resolution details, like drug occupancy and whether the S6 helix is alpha helical or has a pi bulge. The authors even say themselves that they see evidence for a mixed population of alpha helix and pi-bulge in both their EA/PA structures. I highly recommend that the authors use focused classification, whether in cryoSPARC or Relion, ideally with symmetry expansion, to carefully sort out occupancy for drug vs lipid and the helical status of S6 to get a clearer

picture of their states. The authors could consider a similar approach to sorting as used in doi: 10.1038/s41594-024-01299-2, where focused classification could neatly sort very similar features.

We appreciate the reviewer's suggestion regarding the paper by Arnold et al. While we agree that their 3D-focused classification effectively sorted heterogeneity in their study, it did not apply to our case. A fundamental difference lies in the sample preparation: they were able to purify the channel with an empty vanilloid pocket, which is something we are not able to do (and which would also be beyond the scope of our study). In contrast to Arnold et al., where the 3D classification is centred on the presence or absence of density in the pocket (representing clear discrete heterogeneity), our situation always involves an occupied pocket, either by lipid or EA. Additionally, there is significant overlap in the densities of the two ligands, with the only region that doesn't overlap being half of one of the lipid tails. However, this area is flexible and is typically found in a noisy environment.

We acknowledge the reviewer's point about the heterogeneity of specific domains in our data sets. However, in the case of fully occupied EA (TRPC5:EA^{PA}), this heterogeneity is not of a discrete compositional nature (such as the presence of lipid or EA) but is rather a continuous conformational one. This helps explain the relatively poor resolution of the π-bulge. Regardless of the masks employed (**Figure R1c,d,e,g** – see below), our results consistently indicated full occupancy. While it is possible that there are minimal partially occupied binding sites, we were unable to sort out lipid-occupied particles. Throughout our classification attempts, we experimented with various initial low-pass structures, ranging from 4 Å to 15 Å in increments of 2 Å. Each mask was applied in tight, loose, and very loose configurations, with soft padding of either 3, 6 or 12 pixels. Each classification was derived from the 3D classification, and the focused classification process was evaluated based on both the raw output map and after local refinement without alignment or symmetry. The masks illustrated below serve to exemplify the regions that were masked; they do not represent an exhaustive summary of all the classification and sorting tasks undertaken in the current study.

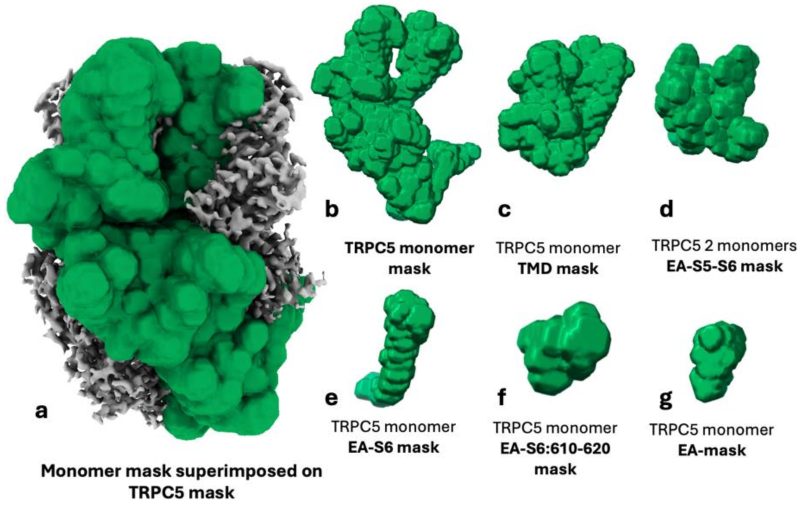

Figure R1. Examples of masks used for 3D focused classification. a) Mask (green) covering one TRPC5 monomer superimposed on TRPC5 cryoEM map; b) TRPC5 monomer mask; c) mask covering the transmembrane domain of a TRPC5 monomer; d) mask covering the S5-S6 helices and EA from two TRPC5 monomers; e) mask covering a S6 helix and one EA molecule; f) mask covering one EA molecule and residues 610-620 found on the S6 helix; g) mask covering one EA molecule.

We agree with the reviewer that achieving a better separation of the mixed population of alpha helices and π-bulges in the TRPC5:EA^{PA} data sets would be desirable and significantly beneficial for a clearer understanding of channel dynamics. Although we explored various approaches to address this issue, it turned out to be a challenging task. Unfortunately, regardless of the methodologies we employed, we could not achieve a clear distinction between TRPC5:EA^{PA} structures containing π-bulges and those with alpha helices. Among our attempts, we applied symmetry expansion of each state followed by focused classification with masks that covered either the EA and the S6 helix (**Figure R1e**) or the EA and three residues above and below the π-bulge (**Figure R1f**). We experimented with numerous classification parameters, adjusting each mask between tight, loose, and very loose configurations, with soft padding of either 3, 6 or 12 pixels. Additionally, we attempted a binary approach where each of the two states was symmetry-expanded and classified into two classes using focused classification with a mask covering the EA and the adjacent S6 helix (**Figure R2**). This strategy was applied twice in a sequential manner, with refinement of each class in between through local refinement. Despite these strategies, the π-bulge and alpha-helix structures could not be separated, indicating a strong continuous flexibility. We have already acknowledged this limitation in our study of TRPC5:EA^{PA} and presented the two different conformations derived from our second dataset (without PA), which clearly illustrates the two states we believe are essential for understanding protein dynamics and which highlights the challenges involved in such studies.

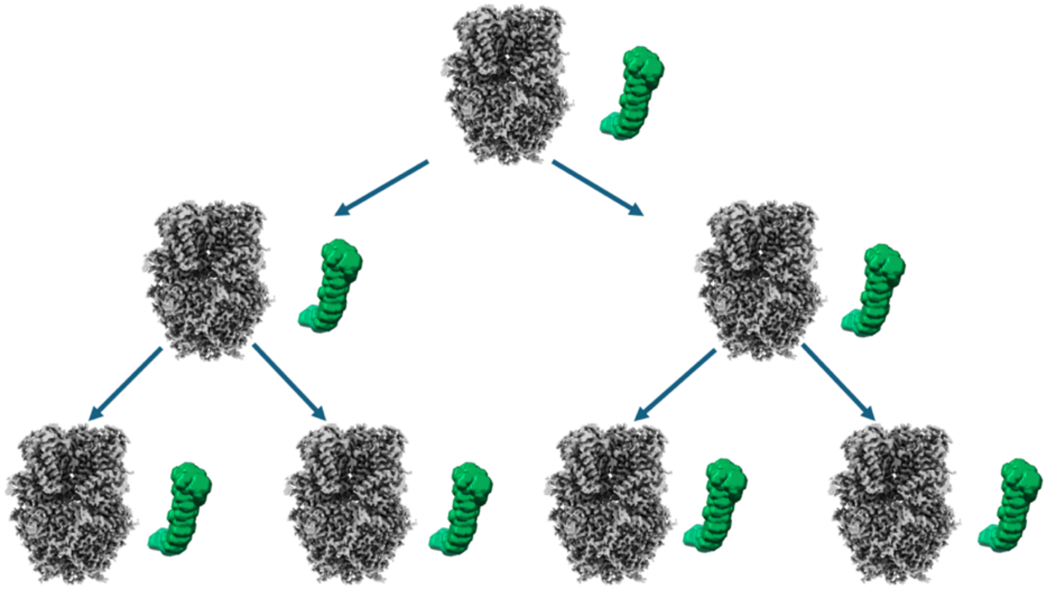

Figure R2. Binary 3D classification strategy.

Examples of focused classification attempts for both states of TRPC5:EA^{PA} are illustrated in **Figure R3**. We chose to include these here to demonstrate the continuous transitional heterogeneity between π-bulges and alpha-helices, alongside the full occupancy of the EA binding site (with no lipid density). Since the used mask covers both the EA and the S6 helix, it provides the best representation.

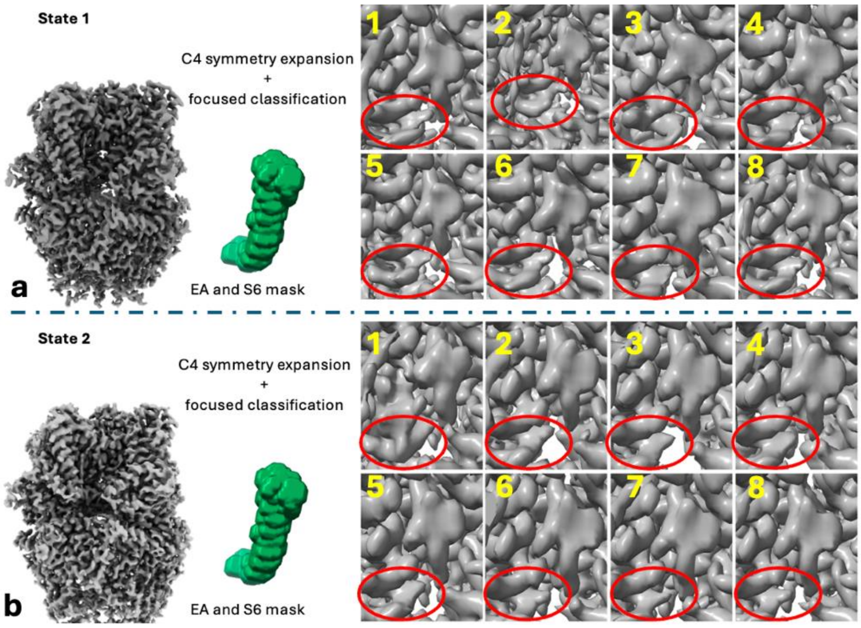

Figure R3. Examples of results focused 3D classification on symmetry-expanded particles from both states of TRPC5:EA^{PA} employing a mask covering one molecule of EA and the adjacent S6 helix. a) TRPC5 in state 1 and the focused classification mask. **a1-8)** cryoEM density of EA and adjacent helices from two class resulted after 3D focused classification of TRPC5 state 1 (red circle showing the π-bulge region); **b1-8)** cryoEM density of EA and adjacent helices from two class resulted after 3D focused classification of TRPC5 state 2 (red circle showing the π-bulge region).

Although 3D classification methods are effective at distinguishing discrete clusters of particle subpopulations, they often struggle in scenarios involving conformational heterogeneity or continuous transitions. For this reason, we used 3DVA, which takes a different approach by not relying on prior information. Instead, it utilises eigenvector analysis to resolve subtle components amongst the particles in the dataset. As all our attempts to classify and sort the particles of TRPC5:EA^{PA} indicated full EA occupancy at the four binding sites, we decided to proceed with the results from 3DVA, which enabled us to successfully cluster various TRPC5 states and, in our second data set without PA, TRPC5:EA stoichiometries. We did not include all the sorting attempts because, despite our extensive efforts, they proved unsuccessful.

At the reviewer's suggestion, we have included the mask used for 3DVA in the revised cryoEM workflow (**Extended Data Figures 2,8**).

b. I also encourage the authors to carefully consider symmetry in the models where it is applied. There is clear indication of multiple conformations at the pi-bulge in both their EA/PA structures, but also at the bottom of S6 in their EA 4:4 structure. This may be due to heterogeneity that can be sorted out, but it could also be due to C4 averaging of particles that have subtle asymmetry.

Each map was initially refined without applying symmetry (**Supporting Note 2**). This was followed by a refinement using C4 relaxed symmetry with maximisation enabled, where only the single best pose was retained for reconstruction. Finally, the maps that displayed similar features across all four monomers – evaluated based on the density in the binding site and the S6 helices – were refined. Specific symmetries were imposed: C4 symmetry for full

occupancy, C2 symmetry for maps with a 2:2 stoichiometry. In cases of mixed occupancy (where stoichiometry could not be determined), we used C4 symmetry in relaxed models.

Model Building:

c. The authors have relied on Model Angelo to build their initial models; Model Angelo can be a good starting point, but it is not an infallible tool. The authors seem to have trusted it to build tricky/heterogeneous portions of the model rather than using better data processing approaches to resolve those areas of heterogeneity. For example, the bottom of the S6 helix is pretty messy in many of their structures, but frequently has residues modelled in despite that uncertainty. Additionally, there are some areas without heterogeneity that are obviously incorrect, such as the 400-405 loop in the PA only model, that could be easily fixed manually.

We agree with the reviewer that ModelAngelo serves only as a starting point for building the protein model. As stated in the Materials and Methods section, we used ModelAngelo for initial modelling, followed by manual curation. After this initial curation, our primary focus was on the EA binding site, adjacent helices, and pore helices.

We thank the reviewer for highlighting some errors that have occurred in this process; these have now been corrected.

Regarding the use of improved data processing approaches, we described above how we explored various methods to better resolve these regions. This included 3D classification, focused classification with and without symmetry expansion, as well as trying different masks and local refinements. Unfortunately, further sorting of the particles is limited by factors such as continuous heterogeneity and flexibility caused by compound binding, along with a limited number of particles. The heterogeneity at the base of the S6 helix is particularly pronounced in maps derived from the second dataset, especially those with mixed occupancy, which we did not discuss in the paper. We have now removed all regions that could not be confidently modelled in these maps and have updated **Extended Data Tables 2** to reflect the missing residues. We have also updated the PDB submissions (PDB ID: 9RRF, 9RRQ, 9RVV, 9RRO, 9RRU, 9RSH, 9RSG) and the accompanying statistics in **Extended Data Table 1**.

d. There are a couple of places in the paper where the authors discuss a novel local conformation they observe in their models but that region of the model is either not the best interpretation of the density (such as the register of the bulging loop at the base of S6 in the chain D of the EA 4:2 S1 structure) or one of a couple different conformations that could fit in clearly heterogeneous density (such as the bottom of S6 in the EA 4:4 structure). If the authors want to make a story about a novel conformation they observe in their data, they need to first make sure the region is not heterogeneous (or sort further if it is) and then fit the model with great care.

We thank the reviewer for identifying the mistake regarding the bulging loop; we have corrected it in the PDB model (9RRQ). We enhanced the density of the map by performing symmetry expansion and conducting several focused 3D classifications. While masking two adjacent binding sites (one lipid and one EA) along with the surrounding helices, we observed that a small percentage of particles (<15%) was misaligned, even though they shared the same conformation (**Figure R4**). To address this, we manually aligned the particles by rotating them 90° using volume tools in CryoSparc and then conducted a local refinement, which

resulted in an improved map quality and resolution (see **Extended Data Figures 8,9**). We have updated the deposited map (EMD-54193). Since this issue was due to alignment rather than a new conformation or sorting step, we believe that this adjustment does not need to be incorporated into the workflow.

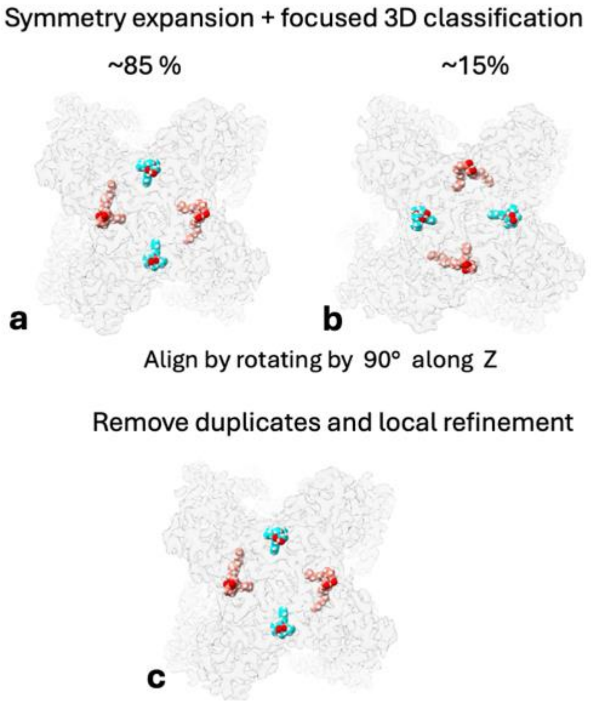

Figure R4. Exemplification of initial misalignment of particles on TRPC5:EA$_{4:2}$-S1 data set. a) frontward orientation; b) backward orientation; c) resulting map after realignment and local refinement.

In the case of the TRPC5:EA$_{4:4}$-S1 conformation, we concur with the reviewer that the area at the bottom of the S6 helix exhibits significant heterogeneity. This heterogeneity arises from the flexibility of the bulging loop that connects the S6 helix to the TRP domain, which (as we have previously shown in **Figure 7**) undergoes rearrangement in certain instances (e.g., in case of EA:lipid 2:2 stoichiometry). Due to the high flexibility and lower resolution of this region, we acknowledge that multiple conformations can be modelled. However, our assignment of a new protein conformation is grounded in the observable changes in the pore-forming helices (which exhibit good map quality) and in the lower gate. This model is better supported by the map than the apo model (see **Figure R5** below). Following a focused 3D classification with symmetry expansion and masking one EA and surrounding helices, we discarded approximately 10% of particles that did not show obvious EA density. After removing duplicates and conducting local refinement, both the map quality and resolution improved slightly (approximately 0.01 Å). We have updated our cryo-EM workflow to reflect this step (**Extended Data Figure 8**). We have also updated the deposited map (EMD-54189).

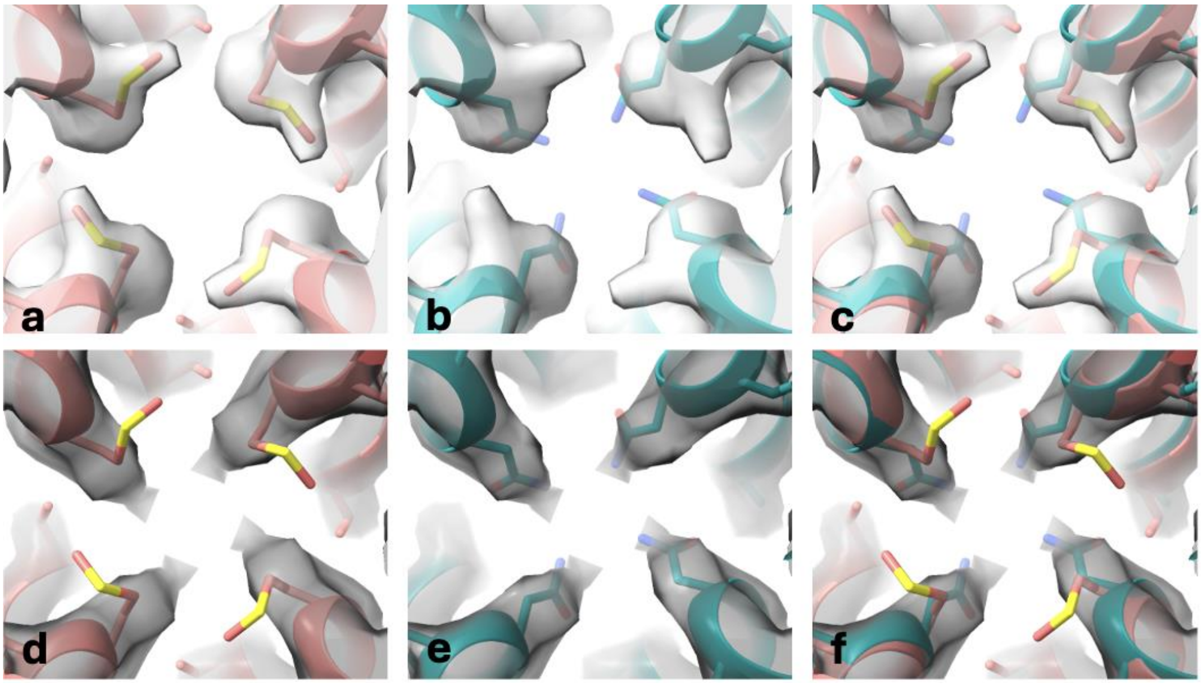

Figure R5. Comparison of lower gate cryoEM map and model of TRPC5:EA$_{4:4}$-S1 and TRPC5:EA^{PA} -S1. a) CryoEM map and model of lower gate from TRPC5:EA$_{4:4}$-S1(red), b) lower gate of TRPC5:EA^{PA}-S1(cyan) model fitted in the TRPC5:EA$_{4:4}$-S1 cryoEM map; c) superimposed lower gate of TRPC5:EA$_{4:4}$-S1, and TRPC5:EA^{PA}-S1 model fitted in the TRPC5:EA$_{4:4}$-S1 cryoEM map; d) cryoEM map and model of lower gate from TRPC5:EA^{PA}-S1 (red), e) lower gate of TRPC5:EA^{PA} -S1 (cyan) model fitted in the TRPC5:EA$_{4:4}$-S1 cryoEM map; f) superimposed lower gate of TRPC5:EA$_{4:4}$-S1, and TRPC5:EA^{PA}-S1 model fitted in the TRPC5:EA^{PA}-S1 cryoEM map.

Minor Issues:

3. Many of the structural figures were difficult to interpret or sometimes even mis-represented the actual structures. I found that I needed to go to the PDB structures to figure out where the residues/binding pockets the authors discussed were rather than being able to use the provided figures.

    Examples:

    a. Figure 4a-c (and subsequent figures of a similar style) were particularly difficult to interpret as the clipping from the depth cuing cut off the helix that F520 is attached to. Additionally, the authors do not label the helices (eg, S5, S6) in many of their figures or mention in the figure legends which helices are depicted in the figures.

We thank the reviewer for this comment. We have changed the style of figure panels showing the aromatic interaction networks of Phe520 (i.e. **Figure 4a-c**; **Figure 5c,d,f**), showing the helices as partially transparent ribbons along with the labelling of the two helices. We provide an example in **Figure R6**. We consider these changes to provide a much clearer interpretation of the Phe520 interaction and dynamics.

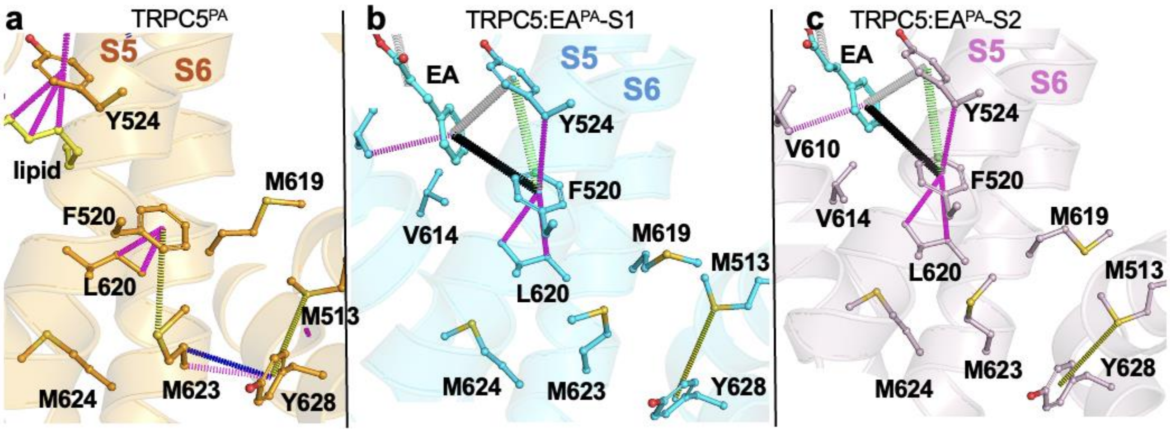

Figure R6. New style of Figure 4a-c.

b. The helix-as-tube representation created odd representational artifacts in figures 5e and 7f that misrepresent the actual conformational changes the authors observed. So Figure 7f, for example, shows a complete dislocation of the ends of two helical segments, when a coil representation shows that the actual conformational change is a modest bend caused by a pi bulge (compare 7f to 7p). I think the authors would be far better served by completely skipping the helix-as-tube representation and make backbone comparisons with a simple coil or ribbon representation.

We agree with the reviewer and have updated the figures to use ribbon representations of the helices. This change was made in **Figure 7e,f,g,I,j,k**. The new panels of **Figure 7** are shown in **Figure R7**.

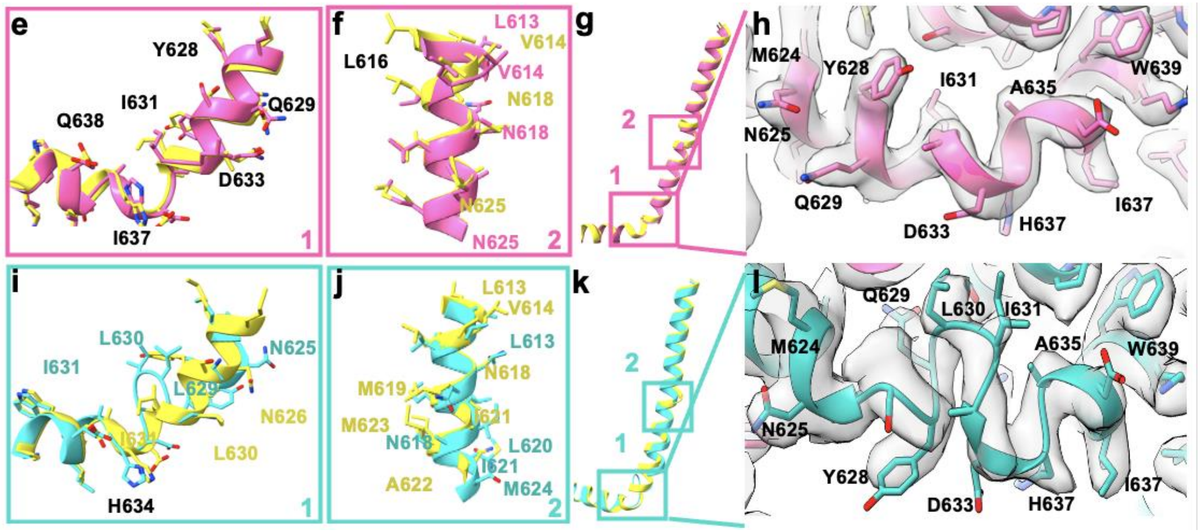

Figure R7. New panels of Figure 7e,f,g,i,j,k illustrating the helices as ribbons instead of tubes.

c. Since the authors are presenting F520 as a key residue for EA signal transduction, it would be very helpful if the authors could make a figure clearly

comparing the three conformations for it they observe: Apo/PA, EA/PA, and the third conformation oriented out of the pore.

We have added a new figure (**Extended Data Figure 11** – see below) were we show a direct comparison of the three conformations of Phe520.

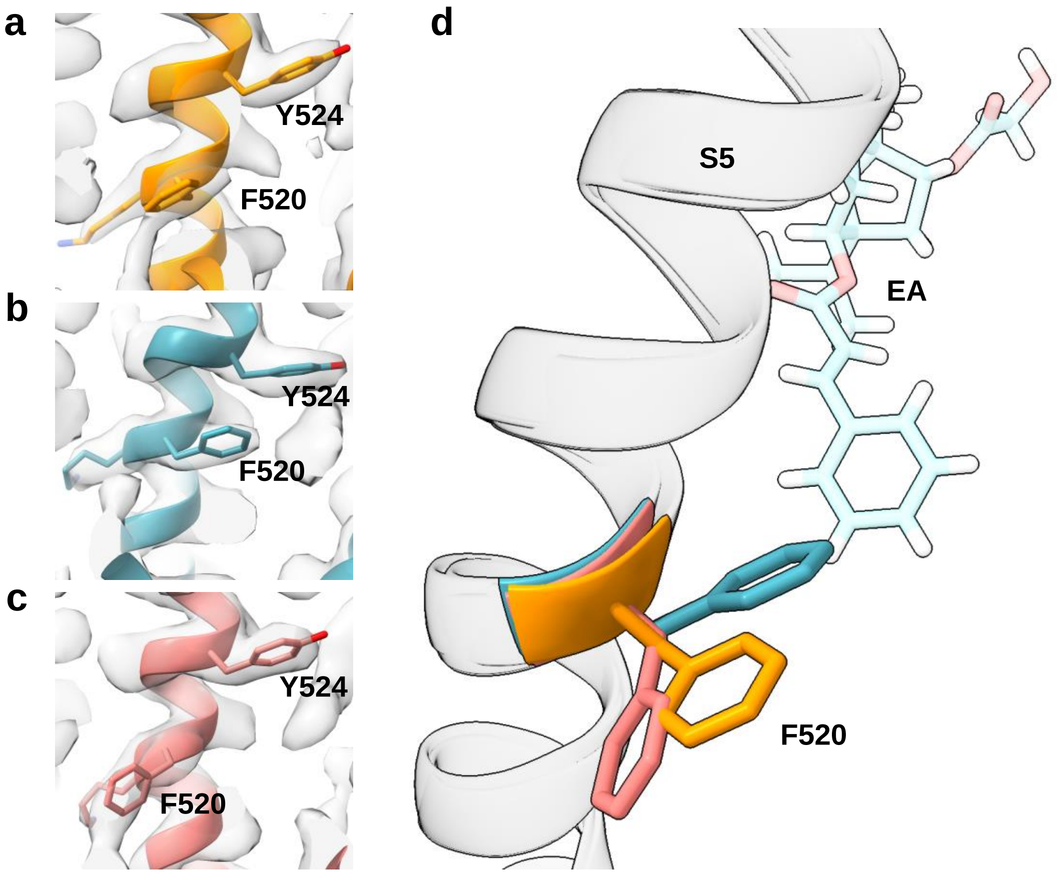

Extended Data Figure 11. Dynamics of Phe520 in TRPC5 activation. a, TRPC5^{PA}. **b,** TRPC5:EA^{PA}-S1. **c,** TRPC5:EA$_{4:4}$-S1. **d,** Superimposition of S5 helix from the three structures showing different orientation of Phe520.

4. The model provided for the PA only map is very slightly offset from the map - double check this in what you have deposited to the RCSB.

We apologise to the reviewer for submitting an outdated version of the model, which also resulted in incorrect modelling of the good density (n400-405). The model uploaded to the PDB was centred correctly.

5. The pore profiles in extended data figure 11o don't seem to quite match up with the pores depicted. Specifically, the blue and yellow lines (corresponding to the two 4:2 conformations) are show as having very narrow selectivity filters, narrower than the key constriction diameter preventing the passage of water, while the other structures have much looser selectivity filters. Examination of the panels g,h,k and l, which show the 3D shape of the pore, are not consistent with that measurement.

We thank the reviewer for identifying the inconsistency between pore shape and measurements, which helped us avoid an awkward mistake before publication. We corrected this in the revised version (see below; now **Extended Data Figure 12O**).

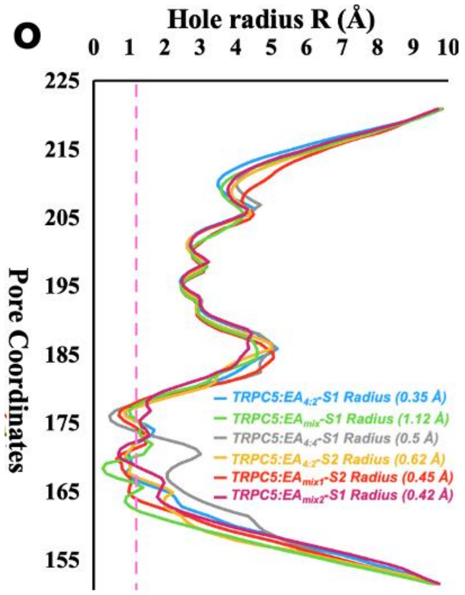

Corrected Extended Data Figure 12o (previously Extended Data Figure 11o).

Reviewer 2

In this report, Porav and colleagues describe several (eight) new cryo-EM structures of human TRPC5 bound by its agonist Englerin A (EA). Although multiple high-resolution TRPC5 structures, including several ligand-bound ones, have been made available, the EA-bound TRPC4 or TRPC5 structures have been proven to be difficult to obtain. Here, the authors tried two new strategies to preserve EA binding to TRPC5 during the sample preparation and succeeded in resolving the EA-bound structures, which have been long overdue, at excellent high resolutions (2.4-3.2 Å). Although they were unable to obtain an open structure for TRPC5 channels, the resolved intermediate and/or desensitized states are valuable for the understanding of TRPC5 structural dynamics and gating mechanisms, as well as the rationale design of drugs targeting TRPC5 and related channels. This work is significant because TRPC channels are strongly implicated in many physiological functions and diseases. The cryo-EM structures are of high quality, and functional studies are thorough and informative.

We thank the reviewer for their detailed assessment of our manuscript and for their insightful questions and for catching several typos and errors of phrasing.

The following suggestions may help improve the manuscript.

1. The title of the paper seems to suggest that a Met-aromatic motif plays a critical role in TRPC5 channel activation. However, this point is not clearly presented in the Results section. Although S/π interactions are mentioned in several places, which particular

S/π interaction is considered to be critical for channel activation is not disclosed. I suggest that the authors dedicate a specific section to present the data on this Met-aromatic motif and its function. Otherwise, the title should be modified to tone down the emphasis on this motif.

Our detailed structural analysis of TRPC5:EA complexes at different stoichiometries reveal a number of interesting molecular rearrangements of TRPC5 induced by EA binding. The most remarkable of these involves the aromatic interaction network around Phe520, which seems to act as an important switch in TRPC5 activation (also consistent with our structural work on other activators: Müller et al. 2024, https://www.biorxiv.org/content/10.1101/2024.07.12.602451v1, now accepted for publication in Nature Chemical Biology).

While we have studied a large number of TRPC5 variants to understand the effect of mutations on EA-induced TRPC5 activation, our main manuscript focuses on the role of aromatic residues Phe520 and Tyr524 in the EA binding site (**Figure 2**), and the aromatic interactions around these residues (**Figure 4** [for the 'PA' data set] and **Figure 5** [for the 'non-PA' data set]). This includes a relatively understudied Met-aromatic motif that has recently been described in other ion channels (as well as other proteins). We have dedicated a major section of our manuscript text to this topic (Results section **'The aromatic interaction network'** and a substantial part of the Discussion section).

To clarify the importance of the wider aromatic interaction network (instead of the focus on Met-aromatic interactions per se) we have now changed the title of our manuscript to: ***"(-)-Englerin A binds a conserved lipid site of TRPC5 and exposes an aromatic interaction network in channel activation."***

2. Pluronic acid (PA) was used to prepare EA-bound TRPC5. Does PA change the apparent affinity and/or efficacy of EA on TRPC5 channels?

As stated in the original manuscript, PA is a *carrier* that is commonly used in pharmacological assays of TRPC1/4/5 channels. While we do not have methods to measure binding affinity of EA to TRPC5, PA does not change the potency of EA at TRPC5. PA does increase the efficacy of EA and the consistency of the EA response. This is most likely related to the poor solubility of EA; PA keeps EA soluble and allows it to reach its binding site in the TM environment. Alternative carriers such as BSA have been used for delivery of EA and other hydrophobic agonists in TRPC1/4/5 assays (see for example Müller et al. 2024, https://www.biorxiv.org/content/10.1101/2024.07.12.602451v1).

3. In many TRP channel structures, the linker that connects CCD to the connecting helix crosses over the CCD of the neighbouring protomer so that its CCD interfaces with the ARD of the adjacent protomer. Is this also the case for TRPC5:EA^{PA}-S1 and TRPC5:EA^{PA}-S2, or only TRPC5:EA^{PA}-S1 but not TRPC5:EA^{PA}-S2?

Yes, this is true only for state 1. In state 2, the CCD loses its interaction with the ARD because the ARD expands simultaneously with the rotation of both the CCD and ARD domains. This is

illustrated in **Extended Data Figure 1**. A similar transition in TRPC5 is described in more detail in Won et al. 2023 (https://www.nature.com/articles/s41467-023-38281-3h).

4. What would be the possible reason for $TRPC5_{F520Y}$ being able to be activated by EA, but not by AM237, BTD, high extracellular Ca^{2+}, and carbachol in combination with Gd^{3+}?

We believe this is related to the differences in their energetic landscapes. While both Phe and Tyr can form similar types of interactions, Tyr is more versatile due to its hydroxyl group, which enables additional interactions, such as hydrogen bonding. Since the other activators are not thought to interact directly with this residue – through π-π or cation-π interactions – tyrosine could stabilise the protein and could favour a closed conformation that requires more energy to disrupt and open the channel.

What would be the possible reason for $TRPC5_{Y524F}$ and $TRPC5_{Y524W}$ to selectively lose voltage-dependent activation (Figure 4d,e)? Since Tyr is present at this position only in TRPC4 and C5, but not in other EA-insensitive TRPCs, one would think that mutations at this site should mainly affect the activation by EA.

We do not have a definitive answer to this question, but we can draw on our previous study, in which we investigated the role of the lipid-xanthine binding L2 site in voltage activation of TRPC5 (Ptakova et al., 2024; https://pubmed.ncbi.nlm.nih.gov/39210039/). We think that mutation of Tyr524 may disrupt the intrinsic channel gating process, not just EA activation. In Ptakova et al. 2024, we demonstrated that depolarising voltage and lipid-pore helix interactions, which include Tyr524, act together to promote channel opening. The residues forming the lower hydrophobic gate of TRPC5 could be affected by mutations or by removal of resident lipids in the L2 lipid recognition site. By comparing voltage-dependent activation between TRPC5 and TRPC4, we showed that the voltage sensitivity of TRPC5 likely originates from a region other than this L2 site.

5. The I-V curve for $TRPC5_{L613A}$ appears linear. Can this be explained based on resolved new structures? As the authors pointed out in the discussion, a previous study has determined Asp633 to be the determinant of the double rectification I-V of TRPC5. Does the L613A substitution change the position of Asp633 in the channel structure?

It is difficult to explain this from a structural perspective. The changes in I-V relationship due to the TRPC5-L613A mutation resemble the effect of neutralisation of Asp633 (by mutation to Asn) in the cytosolic negatively charged ring (Obukhov and Nowycky 2005; https://pubmed.ncbi.nlm.nih.gov/15689561/), although a shallow hump is still visible on the I-V curves of the $TRPC5_{L613A}$ variant. We are not able to predict if the impact of the mutation of Leu613 to Ala can extend to structural rearrangement of Asp633. We reason that, in general, a linear relationship between current and voltage is characteristic of the channel approaching full/saturating activation. It is therefore possible that the mutation increases the degree of

expansion of the lower pore, reducing the electrostatic reach of Asp633 without changing its structural arrangement.

6. Line 4, a reference is needed for galangin modulation of TRPC5.

Thank you for spotting this missing reference; a reference to Naylor et al. 2016 https://pubmed.ncbi.nlm.nih.gov/26565375/ has now been added in line 41.

7. Lines 136-137 state that "The $TRPC5^{PA}$ map displays a well-defined, characteristic lipid U-shape (Figure 1i)". Please comment whether the PA treatment is required for seeing this lipid U-shape.

PA is not necessary to obtain clear lipid U shape density in TRPC5 maps. This lipid density is consistently observed in all published apo structures of TRPC5 (e.g., PDB ID: 7E4T, 7X6C, 8GVW), as well as in other TRPC channels (e.g., PDB ID: 7B0J [TRPC4], 7DXB [TRPC3], 7DXF [TRPC6]). It can also be seen in ligand-bound channels in which the ligand binds to a different site (e.g., PDB ID: 7D4P, 7WDB). Moreover, in our structure of TRPC5:$EA_{\mathbf{4:2}}$-S1, for which no PA was used, two of the four binding sites are occupied by this type of density (**Figure 6a**). The primary role of PA is as a carrier to enhance and maintain the solubility of EA.

8. Lines 206-207, "This result suggests that leucine's bulky, hydrophobic side chain is important for the interaction between TRPC5 and EA". Are you referring to a specific interaction discussed already or the interaction between TRPC5 and EA in a general sense? If for the latter, then are the side chains of Phe, Tyr, Met, Val, etc. discussed in this manuscript less important than that of Leu521? If not, why should Leu521 be specifically emphasized?

We apologise for the confusion. This sentence specifically refers to the effect of the mutation of Leu521 to Ala on the onset of the current. We have amended this sentence in the revised version as follows

"This result suggests that leucine's bulky, hydrophobic side chain at position 521 is important for EA-induced TRPC5 channel gating."

9. Lines 207-208, "$TRPC5_{L521F}$ was constitutively active at negative potentials (Extended Data Figure 4e)". But based on the example shown, it looks like this mutant was constitutively active at both negative and positive potentials. Or does the basal activity represent leak currents?

The currents mediated by $TRPC5_{L521F}$ were significantly different from wild-type TRPC5 only at negative membrane potentials. We do not think that the basal activity represents leak currents because these would also be amplified at positive potentials, which is not the case. At -100 mV, the average current in the absence of any agonist was -0.88 ±0.34 nA in

$TRPC5_{L521F}$ and -0.15 ±0.06 nA in wild-type TRPC5 (P = 0.027; n = 7 and 10). At +100 mV, the outward currents were not significantly different (1.0 ±0.3 nA and 0.6 ±0.2 nA; P = 0.270). Also, the $TRPC5_{L521F}$ currents induced by depolarising voltage at +200 mV (as shown in **Extended Data Figure 4a**) were not significantly different from wild-type channels (**Extended Data Figure 5a**).

10. Line 272, what "engages in an S/π interaction with Met513? Tyr628, Met623, or the C/π interaction between them?

This question refers to the following section:

*"A closer analysis of Phe520 revealed that EA induces changes in its aromatic interaction network[55,56] (**Figure 4**). In the 'apo' structure TRPC5:EA^{PA}, the phenyl ring of Phe520 engages in two types of π interactions: a C/π interaction with Leu620 and a more notable S/π interaction with Met623 (**Figure 4a**). Near Phe520, we also find other ring systems, specifically Tyr524 from the same chain and Tyr628 from the adjacent chain. Examination of the connections between these systems revealed that Tyr524 interacts with the lipid tail through a C/π interaction.* ***Similarly, Tyr628 forms a C/π interaction with Met623, which is involved in the S/π interaction with Phe520 and engages in an S/π interaction with Met513."***

For clarity, we have rewritten the final sentence as follows:

"Similarly, Tyr628 engages in two interactions: a C/π interaction with Met623 and an S/π interaction with Met513."

11. Line 342-343, values for the pore radii should be given for TRPC5:EA^{PA}-S1 and TRPC5:EA^{PA}-S2.

We added the values for the pore radius and reformulated (now: line 344-346) as follows:

*"In comparison to the apo structure TRPC5^{PA} (0.36 Å), the pore radius increases to 0.56 Å in TRPC5:EA^{PA}-S1 and more than doubles to 0.85 Å in TRPC5:EA^{PA}-S2 (**Figure 4g-j**). However, all these structures remain in a non-conductive state, as the expansion is not sufficient for the passage of water molecules or ions."*

12. Line 394 says "In TRPC5:$EA_{4:4}$-S1, the benzyl group of Phe520 is rotated by ~125° compared to TRPC5:EA^{PA}-S1". How does it compare to TRPC5^{PA}?

We have now added an additional figure (the new **Extended Data Figure 11** – see below) comparing the three orientations of Phe520 and included the approximate value in the text line 398 (~55°).

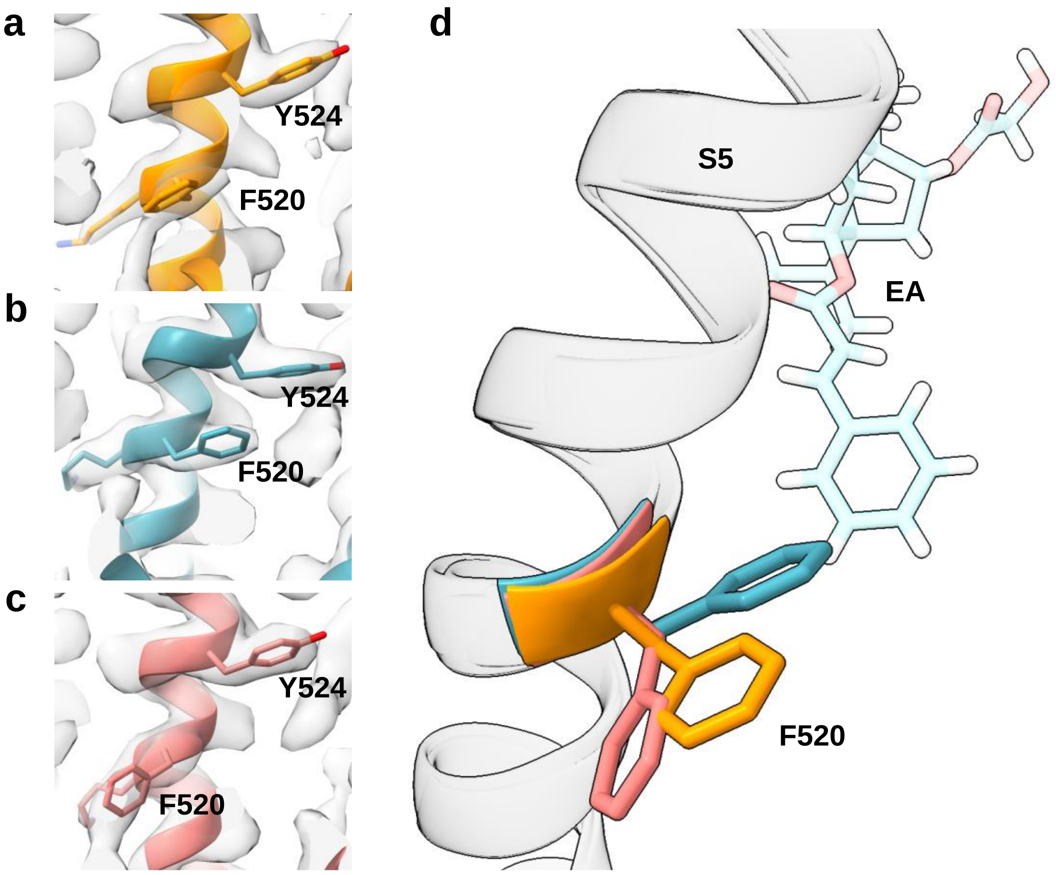

Extended Data Figure 11. Dynamics of Phe520 in TRPC5 activation. a, TRPC5^{PA}. **b,** TRPC5:EA^{PA}-S1. **c,** TRPC5:EA$_{4:4}$-S1. **d,** Superimposition of S5 helix from the three structures showing different orientation of Phe520.

13. Line 469, the second "TRPC5:EA$_{4:2}$-S1" should be "TRPC5:EA$_{4:2}$-S2".

Thank you for catching this; we have corrected this in the revised version.

14. Line 486, "Leu506" should be "Ile506".

Thank you for catching this inconsistency. Leucine 506 is correct. The incorrectly annotated 'Ile506' was corrected in **Figure 5C** and line 489.

15. Line 500, "The disappearance of the S6 π-bulge of subunits I/III..." Should this be "The disappearance of the S6 π-bulge of subunits II/IV…" based on that stated in lines 497-498? Likewise, in line 501, "the positional shift in subunits II/IV…" should be "the positional shift in subunits I/III…".

Thank you. We have corrected this in the revised version.

16. Line 618, “voltage-current relationship” is more commonly expressed as “current-voltage relationship”.

Thank you. We have corrected this in the revised version.

17. In Figure 7b and c, should the pick [pink] helices in these two panels be the same helix at the same position? Then why are the number labels different between them? Also, should L618 in panel c actually be L617?

The reviewer is correct, the regions represent the same position. The helices are not identical because we show a shift at level of Leu613 that is further continued downstream. The confusion resulted from a mistake in labelling of residues which has now been corrected.

18. In Extended Data Figure 6g-j, for ease of reading, the color and style of the symbols should be kept consistent for the same drug concentration in these four panels.

This colour coding is now consistent in the revised version of this figure.

Reviewer 3

The manuscript by Sebastian A. Porav et al. reports a series of high-resolution cryo-EM structures of human TRPC5 in various functional states upon binding to the natural product agonist (-)-englerin A (EA). By integrating structural, biochemical, and mutagenesis approaches, the authors elucidate the EA binding site, identify key functional residues, and propose mechanistic insights into EA-mediated TRPC5 activation. This study offers important contributions to our understanding of TRPC channel regulation and provides a structural framework for rational drug design targeting the TRPC1/4/5 subfamily. While the study is of high interest and presents solid experimental data, several key issues require clarification or further elaboration to strengthen the manuscript's impact and rigor.

We thank the reviewer for their detailed assessment of our manuscript and for their insightful questions and constructive suggestions.

Major Comments:

1. Chen et al. (DOI: 10.1002/pro.70218) have recently reported cryo-EM structures of TRPC5 bound to EA. The authors should cite this work and provide a side-by-side comparison of key structural features. It is essential to clearly articulate the novel aspects and technical advantages of the current study relative to that prior work.

Thank you. The paper by Chen et al. appeared while our manuscript was under consideration. In our revised manuscript, we now cite and discuss this study and compare overall structures and EA binding sites in the new **Extended Data Figure 13** (see below).

The high-quality structures of the TRPC5:EA complex by Chen et al. are consistent with our data sets in the presence of PA, which provides another confirmation that PA does not affect the TRPC5 structure and merely acts as a carrier to enhance and maintain EA solubility.

Our manuscript provides substantial additional insights into molecular interactions between TRPC5 and EA and rearrangements of TRPC5 transmembrane helices and residues upon ligand binding, through detailed structural analysis and functional studies of a large number of TRPC5 variants. In particular, we characterise the functional consequences of mutations of residues surrounding the EA binding site, thereby demonstrating the importance of methionine-aromatic motifs and the wider aromatic interaction network in the structure and function of the channel. This aspect is described in detail in lines 269-318 and discussed in lines 641-656. Moreover, the structures resulting from our additional data set show the effect of protein:ligand stoichiometry on channel conformations and allow the direct comparison of molecular rearrangements of single subunits within channel tetramers depending on the bound ligand (lipid or EA) in the neighbouring site (see for example TRPC5:$EA_{4:2}$-S1 and TRPC5:$EA_{4:2}$-S2).

We have added the following text to the **Discussion** section:

"While our original manuscript was under consideration, Chen et al. published cryoEM structures of TRPC5 in complex with EA[63]. Their structures, which were obtained without the

*addition of PA, are fully consistent with our TRPC5:EA^{PA} structures. A comparison of overall structures and EA binding sites (**Extended Data Figure 13**) provides additional confirmation that the carrier PA does not significantly affect overall TRPC5 structure, nor interactions between TRPC5 and EA."*

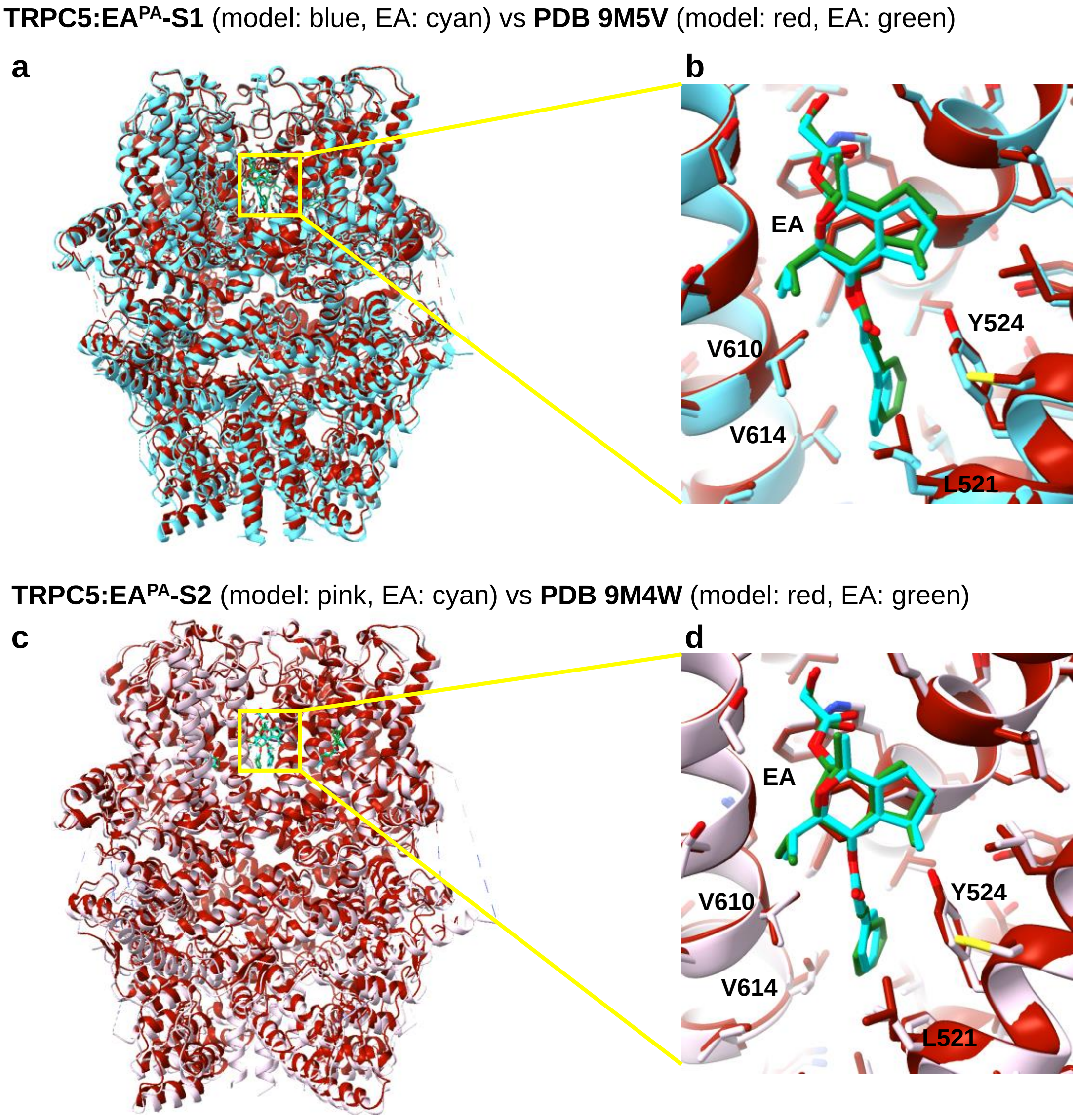

Extended Data Figure 13. Comparison of our TRPC5:EA^{PA} structures to recently reported TRPC5:EA structures by Chen et al.[63] **a**, Overlay of TRPC5:EA^{PA}-S1 (model: blue, EA: cyan) and PDB 9M5V (model: red, EA: green). Both models represent TRPC5 in state 1. **b**, Close up of the EA binding sites in (a). c, Overlay of TRPC5:EA^{PA}-S2 (model: blue, EA: cyan) and PDB 9M5V (model: red, EA: green). Both models represent TRPC5 in state 2. **d**, Close up of the EA binding site in (c).

2. The authors report multiple EA-bound and apo structures, such as TRPC5:EAPA-S1, TRPC5:EAPA-S2, and TRPC5PA. Although the overall architecture appears similar,

the manuscript should provide pairwise RMSD values between these conformations to quantify structural differences.

Thank you for this suggestion. We now provide pairwise comparison of all our structures in the new **Extended Data Table 3**.

	TRPC5^{PA}	TRPC5:EA^{PA}-S1	TRPC5:EA^{PA}-S2	TRPC5:EA$_{4:2}$-S1	TRPC5:EA$_{4:4}$-S1	TRPC5:EA$_{mix}$-S1	TRPC5:EA$_{4:2}$-S2	TRPC5:EA$_{mix1}$-S2	TRPC5:EA$_{mix2}$-S2
TRPC5^{PA}		0.63 Å	0.53 Å	0.69 Å	0.59 Å	0.65 Å	0.52 Å	0.52 Å	0.6 Å
TRPC5:EA^{PA}-S1	1.4 Å		0.47 Å	0.66 Å	0.68 Å	0.65 Å	0.72 Å	0.64 Å	0.63 Å
TRPC5:EA^{PA}-S2	0.6 Å	1.5 Å		0.69 Å	0.71 Å	0.70 Å	0.64 Å	0.68 Å	0.55 Å
TRPC5:EA$_{4:2}$-S1	2.9 Å	1.6 Å	2.1 Å		0.44 Å	0.51 Å	0.76 Å	0.51 Å	0.69 Å
TRPC5:EA$_{4:4}$-S1	2.4 Å	0.9 Å	2.2 Å	1.6 Å		0.45 Å	0.63 Å	0.51 Å	0.59 Å
TRPC5:EA$_{mix}$-S1	2.2 Å	0.8 Å	2.2 Å	0.8 Å	0.7 Å		0.70 Å	0.57 Å	0.52 Å
TRPC5:EA$_{4:2}$-S2	1.7 Å	1.5 Å	1.2 Å	1.2 Å	1.6 Å	1.4 Å		0.55 Å	0.65 Å
TRPC5:EA$_{mix1}$-S2	2.08 Å	2.1 Å	1.6 Å	1.3 Å	1.2 Å	1.3 Å	2.1 Å		0.52 Å
TRPC5:EA$_{mix2}$-S2	2.6 Å	2.3 Å	2.1 Å	2.7 Å	2.4 Å	1.0 Å	0.7 Å	0.9 Å	

Extended Data Table 3. Root mean square deviations of the TRPC5 models. Global RMSD across all atom pairs in purple; global RMSD between pruned atom pairs in blue**.**

3. Figures 3a and 3b detail residue-level conformational shifts around the EA binding pocket. However, the manuscript lacks a comprehensive view of global pocket dynamics. The authors are encouraged to present comparative structural analyses (apo vs. EA-bound) of the full EA binding pocket before focusing on local residue movements. This will significantly improve mechanistic clarity.

Thank you for this suggestion. We have now added two new images (panels d,e) to the revised **Figure 3** (see below), which show the EA binding site with the superimposition of the TRPC5 apo and EA models. Panel d shows a front lateral view of EA and superimposition of apo TRPC5^{PA} model with TRPC5:EA^{PA}-S1 while panel e illustrates a backward lateral view of the sample superimposition.

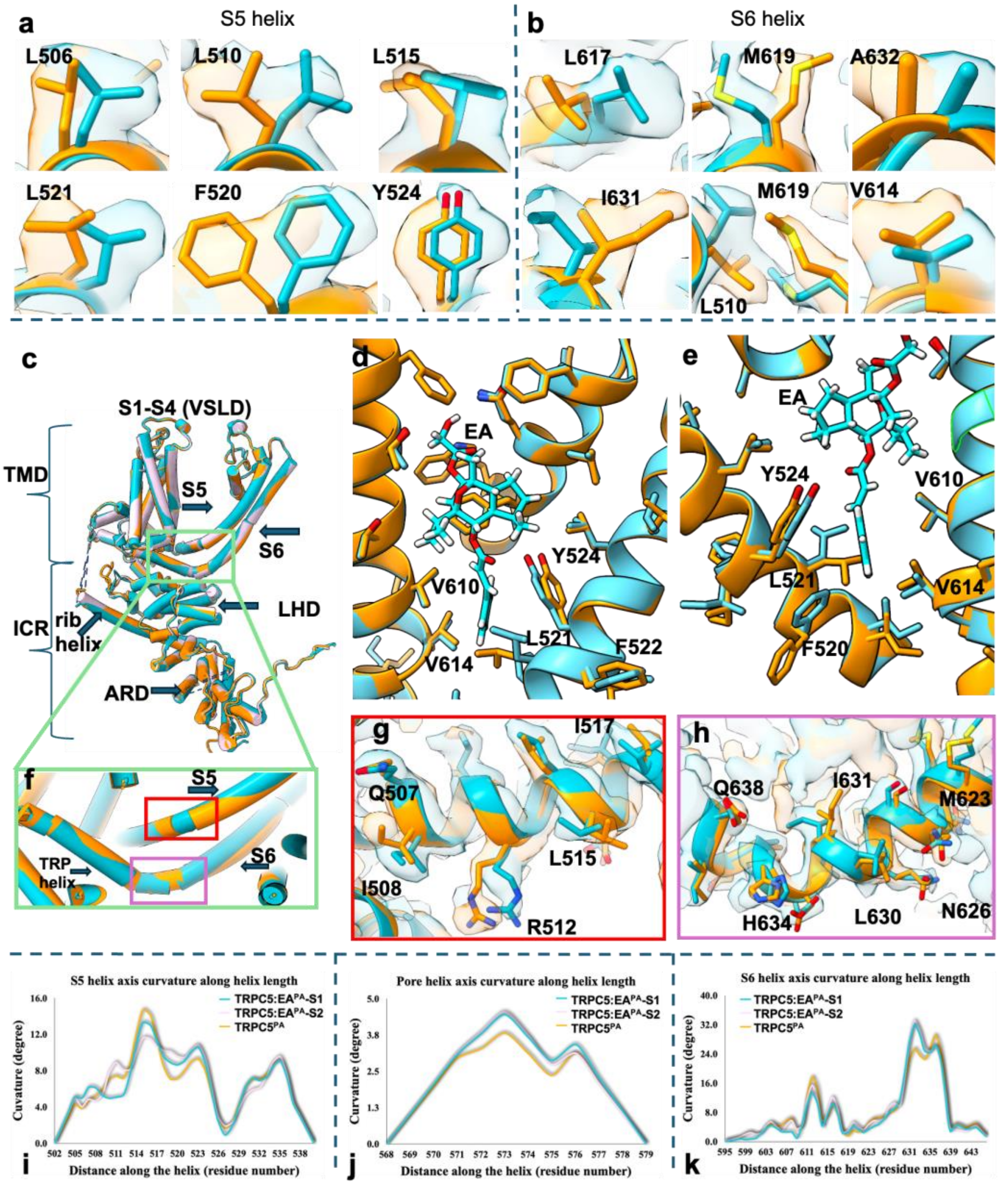

Figure 3. EA binding induces conformational changes of TRPC5 residues and helices. a-b, Close-ups of superimposed maps and models TRPC5:EA^{PA}-S1 (cyan) and TRPC5^{PA} (orange) showing conformational changes of TRPC5 residues on the S5 helix and S6 helix, respectively. **c,** Superimposed model of monomers of TRPC5:EA^{PA}-S1 (cyan), TRPC5:EA^{PA}-S2 (pink), and TRPC5^{PA} (orange), highlighting the transmembrane domain (TMD), intracellular cytosolic region (ICR), voltage sensor-like domain (VSLD) composed of transmembrane helices S1-S4, rib helix, linker helix domain (LHD), ankyrin repeat domain (ARD) and the pore helices (S5 and S6). **d-e,** Front and back view of EA binding site in model TRPC5:EA^{PA}-S1 (cyan) superimposed on TRPC5^{PA} (orange) illustrating the main changes in the ligand binding site upon EA binding. For clarity, the lipid in TRPC5^{PA} is not shown. **f-h,** Close-up of domains (maps and models) of TRPC5:EA^{PA}-S1 (cyan) and TRPC5^{PA} (orange) showing the changes in curvature of the S5 and S6 helices. **i-k,** Curvature analysis (using HELANAL-Plus software[54]) of TRPC5 helices involved in the formation of the EA binding site: the S5 helix (g), the pore re-entrant helix (h), and the S6 helix (i).

4. Line 342 states that the pore radius "doubles" or "triples" in EA-bound structures. However, all presented conformations appear to correspond to closed states. The authors should: (1) Clarify how pore radius measurements were obtained. (2) Explicitly acknowledge that the captured conformations are non-conductive. (3)Discuss the

possibility of using molecular dynamics simulations to explore transition pathways toward open states starting from EA-bound conformations.

Thank you for this suggestion. The pore measurements were done using HOLE interpretation in poreanalyzer (https://poreanalyser.bioch.ox.ac.uk) as described in **Materials and Methods** section. We have now included the following sentences (lines 344-346):

*"In comparison to the apo structure TRPC5^{PA} (0.36 Å), the pore radius increases to 0.56 Å in TRPC5:EA^{PA}-S1 and more than doubles to 0.85 Å in TRPC5:EA^{PA}-S2 (**Figure 4g-j**). However, all of these structures remain in a non-conductive state, as the expansion is not sufficient for the passage of water molecules or ions."*

5. At Line 83, the authors note that incubation with EA alone yielded apo structures, prompting the addition of pluronic acid (PA). Please specify the final PA concentration used. Also, clarify how the "TRPC5:EA structures in the absence of PA" (Line 367) were obtained. Were alternative solubilization strategies employed?

Thank you. The final concentration of PA was 0.01%; this detail has now been added in the Material and Methods section.

For structures in the absence of PA, we did not use different solubilisation methods; instead, we added EA at an earlier stage of the sample preparation process. Details are provided in Supporting Note 2 and in the Material and Methods section.

6. Although structurally similar to EA, the analogue A54 does not activate TRPC channels. The authors should provide structural or mechanistic insights to explain this functional divergence, ideally using structural overlays or binding pocket modeling.

The difference in activity of A54 vs EA is fascinating, considering the very minor change in structure (going from an ester carbonyl in EA to an ether CH2 group in A54). We do not have experimental structures of A54 bound to TRPC5, and obtaining these would be beyond the scope of our investigation. Therefore, we have now modelled A54 into the EA binding site of TRPC5 (through molecular replacement and energy minimisation in Coot), and we have illustrated the results in the new **Extended Data Figure 14** (see below). The modelling suggests that EA and A54 would overlap to a very high degree. In addition, there is no clear interaction of the carbonyl group of EA's glycolate group with TRPC5 residues that would explain how A54 could cause a different effect.

The gating of ion channels such as TRPC5 relies on subtle changes in energetics of different channels states resulting in a change of the open state probability of the channel. Therefore, the existence of activators and inhibitors of specific ion channels with near-identical structures is quite common. Multiple examples exist for TRPC5: small changes to xanthine derivatives (sometimes just the addition of a chlorine atom – see for example Pico145 vs AM237; Minard et al. 2019, https://pubmed.ncbi.nlm.nih.gov/31277085/) can switch a TRPC5 inhibitor to a TRPC5 activator. Further examples can be found in Bauer et al. 2020, https://pubs.rsc.org/en/content/articlelanding/2020/cb/d0cb00126k. We have previously attempted mutagenesis approaches to explain such effects, but so far our results have suggested that the difference between activation and inhibition may not be attributable to interactions with single TRPC5 residues (see the supplementary information of our bioRxiv

preprint Müller et al. 2024, https://www.biorxiv.org/content/10.1101/2024.07.12.602451v1, which has now been accepted for publication in Nature Chemical Biology).

It should be noted that the ether group in A54 will have more conformational freedom than the ester group of EA's glycolate, which may affect the stabilisation of different TRPC5 states.

We have added the following text to the **Discussion** section of our manuscript:

*"Modelling of the TRPC5:A54 structure suggests that the poses of A54 and EA in their TRPC5 binding site overlap to a high degree, and that there is no obvious structural reason for differential TRPC5 modulation by these compounds (**Extended Data Figure 14**). Because gating of ion channels such as TRPC5 relies on subtle changes in the energetics of different channel states, the existence of TRPC5 activators and inhibitors with near-identical structures is not uncommon[37,53,64]."*

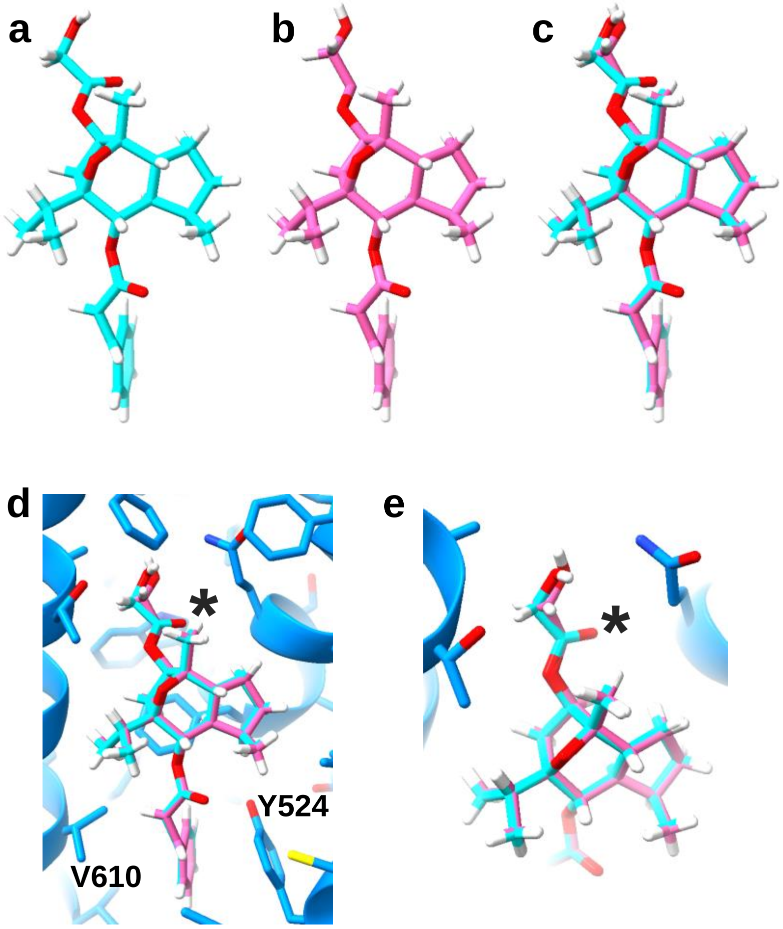

Extended Data Figure 14. Structural comparison of the natural product EA and its synthetic analogue A54. **a,** The TRPC5 agonist EA as bound in TRPC5:EA^{PA}-S1. **b,** The TRPC5 antagonist A54 as predicted to bind to TRPC5:EA^{PA}-S1 (based on molecular replacement of EA and real space refinement in Coot). **c,** overlay of EA (a) and A54 (b). **d,e,** Overlay of EA and A54 in the ligand binding site of the TRPC5:EA^{PA}-S1 structure. The chemical difference between the two compounds (ester carbonyl in the glycolate side chain of EA; ether CH2 group in A54) is indicated with an asterisk.

Minor Comments:

7. Figure 2a–c: Show all interactions within 5 Å. Hydrogen bonds should be depicted with dashed lines rather than solid lines, and this convention should be applied consistently throughout all figures.

Thank you for this suggestion. We have changed the representation of the hydrogen bonds in **Figure 2a-c**, **Figure 5a**, **Figure 6f**, to adhere to convention. We show all EA – protein interactions and we chose to not to extend the radius of interactions as this would only show intra-protein interactions, which could cause confusion.

8. Figure 2e: Maintain consistent axis scales with related panels to clearly highlight the functional loss of Y524A.

We agree with the reviewer that maintaining the same scale should be used for illustrating the functional loss of the mutants ($TRPC5_{Y524A}$ and $TRPC5_{F520A}$). Therefore, we used the same scale to plot the timecourse of average whole-cell currents (left-hand graphs in all panels). On the other hand, we wanted to show whether these non-functional/low-functioning mutants retained at least some residual sensitivity – which can be recognised according to a double-rectifying I/V shape typical of TRPC5. Therefore, we intentionally enlarged the y-axis in the graphs on the right. We believe that this also allows us to compare, for example, the time development of I/V characteristics in TRPC5 variants with altered EA response kinetics (as in panel j).

9. Figures 2d–j: Add a space in "EA (30nM)" for clarity.

Thank you. We have corrected this in **Figure 2d-j**, **Figure 5n** and **Extended Data Figure 4b-l**.

10. Figure 3 vs. Figure 4: Ensure uniform schematic style and illustration conventions across all figures.

We acknowledge importance of consistency between figure style and convention. We have now adjusted the images wherever this was possible. We think the new images show a more consistent style and are more comprehensible.

11. Line 345: Consider modeling alternate conformations for Asn626 as suggested by observed density.

Thank you - we have considered this. We have examined and tried to model Asn626 in a different conformation. However, this is not feasible without additional modifications to the helix, which we cannot assume or implement based on the available data.

12. Extended Data Figure 1: Color labels for TRPC5:EAPA-S1 and TRPC5:EAPA-S2 are reversed.

Apologies - we have now corrected the figure legend to clarify that the overlaps are of all three structures (shown in cyan, pink and orange).

13. Abbreviations: Please add "SBS" to the abbreviations list.

We have now defined SBS as ‘standard bath solution’ in the first instance it was used.

Point-by-point response to reviewers' comments

We thank the reviewers for assessing our revised manuscript and for providing additional constructive comments. We have now made the suggested further changes to our manuscript.

We have included point-by-point responses to the comments below and have provided a highlighted document to show all changes made to the manuscript.

Reviewer 1

In this revised manuscript, Porav et al have implemented reviewer recommendations to improve the interpretability and broaden the perspective of their work. My main comment during the first round of review was to request that the authors perform more exhaustive sorting of the heterogeneous regions of their cryoEM data. I understand that not every dataset cooperates and I appreciate that the authors put effort into this, resulting in two improved maps/models, and explained their process. I maintain my earlier view that this work is high quality and worth publishing, especially with the improvements made after the first round of review.

We thank the reviewer for their time, effort and positive comments, and for highlighting additional points for improvement of our manuscript.

However, there were some important oversights in the assembly of the current submission and I must remind the authors to be very meticulous in updating/assembling the manuscript.

1. The authors need to update the structures/maps in their figures where those structures/maps have changed since the initial submission.

a. This is particularly obvious in Figure 7, where the only change to the figure since the previous version is switching the helix-as-tube representation to a ribbon representation in 7e-f,i-j. However, the TRPC5:EA 4:2-S1 structure has been changed since the previous version, going from a C1 relaxed symmetry refinement to a C2 refinement. This structure now has a different and higher quality map, as well as a model which has changed due to refinement into that map. All panels of this figure must be updated to reflect the final maps and models. Since the authors are no longer comparing the two best chains of an asymmetric object, they should also now compare chains I and II rather than II and II.

Thank you for highlighting this. We have now updated **Figure 7** to ensure that all panels are based on the revised map and model for TRPC5:$EA_{4:2}$-S1.

Figure 7 compares subunits I (identical to III) and IV (identical to II). Because of the symmetry, further comparison of subunits would be redundant.

Comparison of data quality for the trans-membrane domains of the individual subunits in the revised TRPC5:$EA_{4:2}$-S1 structure can be found in the new **Extended Data Figure 10**.

b. The authors did further processing to improve the maps for both the TRPC5:EA 4:4-S1 and TRPC5:EA 4:2-S1 structures, but none of the main figures showing maps for these structures appear to have been updated from the previous version of the manuscript. While I have emphasized Figure 7 above, all figures using the maps/models for these structures need to

be updated to match the RCSB/EMDB deposited structures. This includes Fig 5b, all of Fig 6, Extended Data Figure 10 and Extended Data Figure 12j-l, but the authors themselves should also carefully review all figures to ensure that they are presenting the correct versions of their structures.

We have carefully checked the entire manuscript, and have now replaced the following figures to ensure that all panels reflect the revised structures: **Figure 5**; **Figure 6**; **Extended Data Figure 9**; **Extended Data Figure 12**.

2. In my initial review, I asked the authors about sorting their structures based on the ARD/CCD conformation. The explanation they gave in the response to reviewers was fine, but they made no alteration to explain this point in the text. The authors should add a sentence explaining that the two conformations are not expected to have different EA occupancy or be a result of EA binding and why they chose to use the ARD/CCD conformation to guide state separation.

We have now expanded **Supporting Note 1** to include the motivation for using the ARD/CCD conformation to guide state separation. For clarity, we have reproduced the revised **Supporting Note 1** in full here, with additions highlighted:

*"Our initial TRPC5:EA^{PA} map (2.7 Å; C4 symmetry) displayed poorly resolved cytosolic domains, indicating structural flexibility of these domains. We tested different strategies to sort this heterogeneity in our data, including 3D classification, with and without symmetry expansion, focused classification, heterogenous refinement with different low-pass filtering and resolution limits, and various masking strategies (attempting to sort particles based on the presence or absence of EA). However, in all cases the different classes displayed similar EA density but poorly resolved ARDs/CCD. In contrast, 3D variability analysis (3DVA)[50] in CryoSparc[51] using 'clustering mode' resulted in two well-defined clusters depicting two TRPC5 states (**Figure 1a-f; Extended Data Figure 1a,b,d-g; Extended Data Figure 2**). In state 1 (TRPC5:EA^{PA}-S1), the ankyrin repeat domains (ARDs) are close to, and interact with, the coiled-coil domains (CCDs), resembling previous structures of TRPC5 in detergent (PDB 7E4T)[41] and lipid nanodics (PDB 8GVW)[43] (**Extended Data Figure 1d-g**). In state 2 (TRPC5:EA^{PA}-S2), the ARDs rotate counterclockwise (bottom view) while extending from the symmetry axis, similar to a state of human TRPC5 found in lipid nanodiscs (PDB 7X6C)[43] (**Extended Data Figure 1d-g**). The final 3D reconstructions, applying C4 symmetry, yielded maps of TRPC5:EA^{PA}-S1 and TRPC5:EA^{PA}-S2 at global resolutions of 2.5 Å (**Figure 1; Extended Data Figure 2**). The final map of 'apo' TRPC5^{PA} (depicting ARD state 2) was obtained at 2.4 Å resolution (C4 symmetry) (**Figure 1; Extended Data Figure 1; Extended Data Figure 2**). Further efforts to classify and sort particles from the 'apo' TRPC5^{PA} dataset did not reveal additional states."*

We have also added the following sentence to the main text:

*"We used the ARD/CCD conformation to guide state separation as the best method to address heterogeneity in our data; the ARD/CCD states were not expected to result from EA binding or binding site occupancy (**Supporting Note 1**)."*

3. In my initial review, I recommended that the authors remove all helix-as-tube representations in their figures as they inaccurately exaggerated the differences between structures. While the authors did fix this for Figure 7, they did not fix this in Figure 5e. The authors need to update this figure to remove the helix-as-tube representation.

We have removed the helix-as-tube representation from all figures except for **Figure 3, panels c and f**, where we believe this type of representation significantly enhances the understanding of TRPC5 monomer architecture.

4. I appreciate that the authors trimmed their structures to be a little more conservative about interpreting challenging density. While the authors say in the response to reviewers that they updated Extended Data Table 2 to reflect their altered structures, the table is identical to the table from the previous manuscript before these structures had been trimmed. I can see from the residue count in the updated Extended Data Table 1 that there should be quite a few differences.

Thank you for pointing this out and apologies for this oversight on our behalf. We have now included the correct, revised **Extended Data Table 2**.

Reviewer 2

The revision has addressed the comments raised. The paper is much improved.

We thank the reviewer for their time, effort and positive comments.

Reviewer 3

The authors have nicely addressed the reviewer's concerns with new experiments. The new data are consistent with their conclusion, thus I recommend acceptance.

We thank the reviewer for their time, effort and positive comments.
